# Optimization of multiple tuned mass dampers for vibration control of a nonlinear beam

**DOI:** 10.1038/s41598-026-46499-6

**Published:** 2026-04-17

**Authors:** Abanob Zakaria, Ayman E. Nabawy, Ayman M. M. Abdelhaleem

**Affiliations:** Mechanical Design & Production Dept, Faculty of Engineering, Zagazig University, P.O. Box 44519, Zagazig, Egypt

**Keywords:** Beam, Optimization, Vibration suppression, Nonlinear vibration, Tuned mass dampers, Engineering, Mathematics and computing

## Abstract

This research focuses on optimizing tuned mass dampers (TMDs) to achieve maximum vibration suppression in beam structures. We thoroughly investigate both the linear and nonlinear dynamic behaviors of an Euler-Bernoulli beam system. The complex mathematical model of the coupled beam-TMDs system is precisely developed using Hamilton’s principle. A key contribution of this study is its exploration of the incremental effect of adding absorbers, determining optimal parameters as each TMD is progressively integrated. Another novel aspect is the simultaneous optimization of each TMD’s position, stiffness, and damping coefficient. We utilize the particle swarm optimization (PSO) method to achieve this, with the objective of minimizing the area under the system’s response curve. This provides critical insights into improving the dynamic performance and robustness of both linear and nonlinear beam systems.

## Introduction

Vibration control is essential in structural engineering to ensure safety, serviceability, and longevity under dynamic loads such as wind, earthquakes, and operational excitations. Among various mitigation strategies, Tuned Mass Dampers (TMDs) are widely adopted due to their simplicity and effectiveness. A TMD is a passive vibration absorber consisting of a secondary mass, spring, and damper attached to a primary structure. By tuning its natural frequency to match a target mode of the structure, the TMD oscillates out of phase with the primary motion, absorbing and dissipating vibrational energy and thereby reducing oscillation amplitudes^1–3^.

Frahm^1^ first introduced the dynamic vibration absorber concept for suppressing resonant vibrations. Ormondroyd and Den Hartog^2^ later provided analytical solutions for optimal tuning and damping parameters under harmonic excitation, with Den Hartog^3^ formalizing the theoretical foundation for TMD design. A TMD creates an anti-resonance at the target frequency: when the primary structure resonates, the TMD undergoes large, out-of-phase oscillations, applying an opposing force that reduces vibration while its internal damping dissipates the absorbed energy.

In modern engineering applications, TMDs have been widely implemented in high-rise buildings, long-span bridges, robotic systems, and mechanical installations. Their effectiveness has been demonstrated in full-scale deployments, including supertall buildings under typhoon excitation^4^, curved pedestrian bridges utilizing distributed MTMD configurations^5^, and human-induced excitation scenarios requiring variable-stiffness adaptive TMDs^6^. Moreover, multi-TMD arrangements have been successfully employed for the vibration control of robotic manipulators^7^ and flutter mitigation in aeroelastic systems^8^. These advances illustrate the expanding operational domain of TMD technology beyond classical linear settings.

Despite their proven utility, the performance of conventional TMDs deteriorates when the primary system exhibits geometric or material nonlinearities, modal interactions, or time-varying dynamics. This is particularly relevant for slender beam structures, where nonlinear vibration responses arise due to large deflections, softening/hardening stiffness behavior, and multi-mode coupling effects. Recent research has addressed these challenges through improved absorber–structure modeling and optimization, as seen in multi-body wind turbine models integrating TMDs^9^, integrated damping systems for tall buildings^10^, and comprehensive reviews of TMD technology^11^. In parallel, general analytical studies of absorber–structure interaction on flexible systems^12^ continue to inform optimal tuning strategies across linear and weakly nonlinear contexts.

The emergence of computational intelligence has further expanded the design space of TMD configurations. Machine-learning-assisted tuning^13^, metaheuristic optimization for nonlinear structures^14^, and hybrid absorbers for suppressing self-excited vibrations^15^ highlight the growing role of data-driven and algorithmic approaches. Adaptive and semi-active TMDs employing magnetorheological dampers^16^ or inerter-based architectures^17^ have also been developed to enhance robustness under parametric uncertainties.

In parallel, inerter-based absorbers^18^ and energy-harvesting TMDs utilizing piezoelectric electromechanical coupling^19^ have added new functional dimensions to absorber design, enabling simultaneous vibration reduction and operational energy capture. Artificial-intelligence frameworks, such as ANN-based formulations, have shown promise for rapid prediction of optimal tuning parameters^20^. These developments underscore the broadening landscape of vibration absorbers and the growing demand for advanced optimization methodologies.

Beam-specific vibration suppression has also been studied extensively. TMDs and NES-type absorbers have been investigated for beams subjected to moving loads^21^, rotating-metamaterial behavior^22^, embedded nonlinear absorbers in metamaterial beams^23^, nonlinear cantilever-beam energy sinks^24^, nanobeams with higher-order continuum theories^25^, and optimized TMDs for cantilever-beam response mitigation^26^. Additional studies on multi-TMD arrangements^27^, experimental validation of TMD-based seismic control in scaled steel frames^28^, and damper distribution in shear buildings^29^ demonstrate the effectiveness of multi-absorber approaches under diverse loading regimes. Further, elastic support optimization^30^, nonlinear viscoelastic beam dynamics^31^, multimodal suppression using time-delayed absorbers^32^, parallel NES arrays for Timoshenko beams^33^, TMD-inerter systems under moving loads^34^, magnetic tristable NES absorbers for cantilever beams^35^, and boundary-controlled Euler–Bernoulli beams^36^ collectively reinforce the importance of tailored absorber configurations for advanced beam structures.

However, despite the rich literature on single TMDs, nonlinear absorbers, and distributed damping concepts, there remains a critical research gap in the systematic optimization of multiple tuned mass dampers (MTMDs) for nonlinear beams. Nonlinear beam behavior introduces frequency shifts, amplitude-dependent stiffness, internal resonances, and energy transfer across modes, which can severely compromise the performance of conventionally tuned single TMDs. Multi-absorber configurations offer the potential to address multimodal and nonlinear characteristics more effectively, but optimal damper placement, tuning, mass allocation, and damping distribution remain insufficiently explored—particularly under strong nonlinearities and broadband or nonstationary excitation. Most studies optimize TMD parameters in isolation or focus on either linear or nonlinear systems independently. The concurrent optimization of absorber count, spatial positioning, stiffness, and damping coefficients—and how these parameters interact with internal damping—has not been systematically addressed.

This study advances the understanding of Tuned Mass Damper (TMD) optimization by presenting a systematic comparison of linear and nonlinear beam behavior under multiple TMD configurations. Unlike previous studies that focus on isolated parameters, this work simultaneously optimizes the number of absorbers, their spatial positioning, stiffness coefficients, and damping coefficients. The investigation further examines how internal damping interacts with multiple TMDs to influence vibration suppression and provides detailed response analysis at both the excitation point and the fundamental mode antinode. For each TMD configuration, Particle Swarm Optimization (PSO) was employed to determine optimal positions, stiffnesses, and damping coefficients. To ensure global optimality, the algorithm was executed from 20 different random initial populations. The solutions consistently converged to a narrow range, strongly indicating that the reported results represent a global optimum.

Through rigorous modeling and optimization, this work contributes to a deeper understanding of absorber deployment strategies for nonlinear beam vibration control and advances the design of efficient multi-TMD systems capable of operating effectively within the inherently nonlinear dynamic behavior of slender structural elements.

## Mathematical formulation

**Fig. 1:**
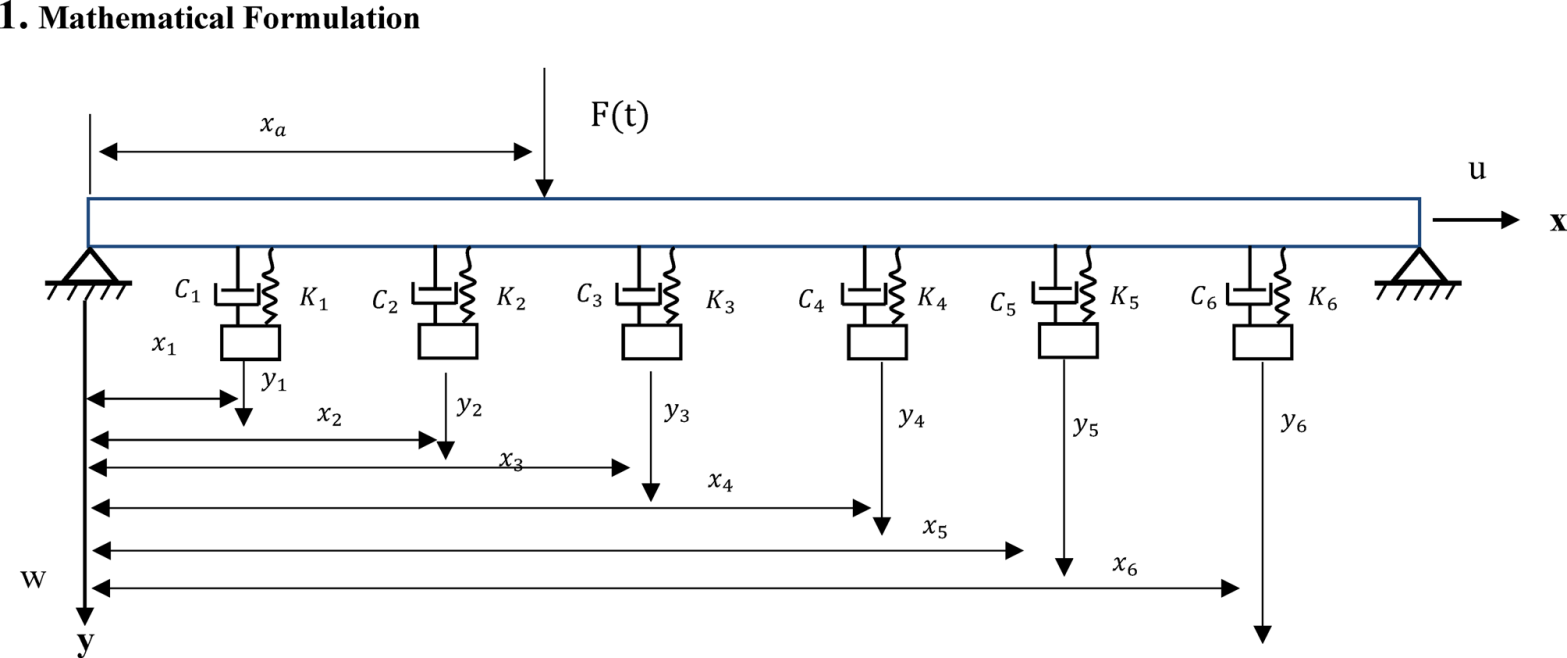
A schematic of a nonlinear beam with multiple tuned mass dampers.

In Fig. 1 the beam is modeled as an Euler–Bernoulli beam with length *L*, and hinged-hinged boundary conditions. The beam’s vertical displacement is denoted as *w* along *y* axis. *u* denotes the displacement component of the beam along *x* axis. A number of Tuned Mass Dampers (TMDs) $\:{N}_{D}$ are attached to the beam each at position $\:{x}_{j}$, consisting of mass $\:{M}_{j}$, damping $\:{C}_{j}$, Linear stiffness $\:{K}_{j}$. The TMD exerts a force on the beam:

1 $$\:{F}_{j}={K}_{j}{w}_{rj}\delta\:\left(x-{x}_{j}\right)+{C}_{j}{\dot{w}}_{rj\:\:}\delta\:\left(x-{x}_{j}\right)$$

$\:{w}_{rj}\:$is the relative displacement between each TMD and the beam ($\:{w}_{rj\:\:}={y}_{j}-{w}_{j}$). $\:{y}_{j}$ is the displacement of the jth absorber and $\:{w}_{j}$ is the vertical displacement of the beam at x= $\:{x}_{j}$. $\:\delta\:\left(x-{x}_{j}\right)$ is the Dirac delta function used to locate the force exerted by each TMD. The beam is under the influence of a time-varying force, *F(t)*.

### Beam kinematics and dynamics

Using von Kármán nonlinear strain theory, the axial strain is^25^:

2 $$\:{\epsilon\:}_{xx}\left(x,y,t\right)=\frac{\partial\:{u}_{0}\left(x,t\right)}{\partial\:x}-y\frac{{\partial\:}^{2}{w}_{0}\left(x,t\right)}{\partial\:{x}^{2}}+\frac{1}{2}{\left[\frac{\partial\:{w}_{0}\left(x,t\right)}{\partial\:x}\right]}^{2}$$

$\:{u}_{0}\left(x,t\right),\:{w}_{0}\left(x,t\right)$ are the displacement components at the midplane of the beam (*y = 0*).

The stress is given by:

3 $$\:{\sigma\:}_{xx}\left(x,y,t\right)=E[{\epsilon\:}_{xx}\left(x,y,t\right)+\eta\:\frac{\partial\:{\epsilon\:}_{xx}\left(x,y,t\right)}{\partial\:t}]$$

4 $$\:{F}_{j}={K}_{j}({y}_{j}-{w}_{0}\left(x,t\right))\delta\:\left(x-{x}_{j}\right)+{C}_{j}(\dot{{y}_{j}}-\frac{\partial\:{w}_{0}\left(x,t\right)}{\partial\:t})\delta\:\left(x-{x}_{j}\right)$$

where $\:E$ is Young’s modulus and $\:\eta\:$ is the Kelvin-Voigt damping coefficient.

Hamilton’s principle for the system is expressed by the variational statement:

5 $$\:{\int\:}_{0}^{t}\left[\delta\:K-\left(\delta\:U-\delta\:W\right)\right]dt=0$$

Here, $\:K$ represents the kinetic energy, $\:U$ the strain energy, and $\:W$ the work done by the applied loads, which are defined as^21^

6 $$\:K=\frac{1}{2}{\int\:}_{0}^{L}{\int\:}_{A}\rho\:\left[{\left(\frac{\partial\:{u}_{0}}{\partial\:t}-y\frac{{\partial\:}^{2}{w}_{0}}{\partial\:x\partial\:t}\right)}^{2}+{\left(\frac{\partial\:{w}_{0}}{\partial\:t}\right)}^{2}\right]dAdx$$

7 $$\:U=\frac{1}{2}{\int\:}_{0}^{L}{\int\:}_{A}{\sigma\:}_{xx}\left(x,y,t\right){\epsilon\:}_{xx}\left(x,y,t\right)dAdx$$

8 $$\:\delta\:W=\frac{1}{2}{\int\:}_{0}^{L}\left[\sum\:_{j=1}^{\:{N}_{D}}{F}_{j}\left(x,t\right)+F\left(t\right)-{c}_{w}\frac{\partial\:{w}_{0}}{\partial\:t}\right]\delta\:{w}_{0}dx$$

Where $\:\rho\:$ is the density of the beam, $\:{c}_{w}\:$ represents the damping coefficient associated with the beam’s motion and *A* denotes the cross-section area.

the equations of motion are derived^21^:

9 $$\:\frac{\partial\:{N}_{xx}}{\partial\:x}={I}_{0}\frac{{\partial\:}^{2}{u}_{0}}{\partial\:{t}^{2}}$$

10 $$\:\frac{{\partial\:}^{2}{M}_{xx}}{\partial\:{x}^{2}}+\frac{\partial\:}{\partial\:x}\left({N}_{xx}\frac{\partial\:{w}_{0}}{\partial\:x}\right)-{c}_{w}\frac{\partial\:{w}_{0}}{\partial\:t}+\mathrm{F}\left(\mathrm{t}\right)+\sum\:_{j=1}^{\:{N}_{D}}{F}_{j}(x,t)={I}_{0}\frac{{\partial\:}^{2}{w}_{0}}{\partial\:{t}^{2}}-{I}_{1}\frac{{\partial\:}^{4}{w}_{0}}{\partial\:{x}^{2}\partial\:{t}^{2}}$$

Where$\:\:{N}_{xx}$​ and $\:{M}_{xx}$ are stress resultants, and $\:({I}_{0},{I}_{1})={\int\:}_{A}\rho\:(1,{y}^{2})dA$.

Assuming the beam’s nonlinear damping and axial inertia are neglected

11 $$\:{N}_{xx}=\frac{{A}_{xx}}{L}{\int\:}_{0}^{L}\left[\frac{1}{2}{\left(\frac{\partial\:{w}_{0}}{\partial\:x}\right)}^{2}\right]$$

12 $$\:{M}_{xx}=-{D}_{XX}\frac{{\partial\:}^{2}{w}_{0}}{\partial\:{x}^{2}}-\eta\:{D}_{XX}\frac{{\partial\:}^{3}{w}_{0}}{\partial\:{x}^{2}\partial\:t}$$

The extensional and bending stiffness coefficients, $\:{A}_{xx}$​ and $\:{D}_{XX}$​ respectively, are defined as follows:

13 $$\:{(A}_{xx},{D}_{XX})={\int\:}_{A}E(1,{y}^{2})dA$$

The model assumes an Euler–Bernoulli beam with immovable ends, subject to the associated boundary conditions.

14 $$\:{u}_{0}\left(0,t\right)={u}_{0}\left(L,t\right)=\dot{{u}_{0}}\left(0,t\right)={\dot{u}}_{0}\left(L,t\right)=0,\:{w}_{0}\left(0,t\right)={w}_{0}\left(L,t\right)$$

Substituting Eqs. (4), (11) and (12) into Eq. (10) yields the nonlinear control model of the beam.

15 $$\begin{aligned} \:I_{0} \frac{{\partial \:^{2} w_{0} }}{{\partial \:t^{2} }} + c_{w} \frac{{\partial \:w}}{{\partial \:t}} + \eta \:D_{{XX}} \frac{{\partial \:^{5} w_{0} }}{{\partial \:x^{4} \partial \:t}} + D_{{XX}} \frac{{\partial \:^{4} w_{0} }}{{\partial \:x^{4} }} + \left[ {\frac{{ - A_{{xx}} }}{{2L}}\int \: _{0}^{L} \left( {\frac{{\partial \:w_{0} }}{{\partial \:x}}} \right)^{2} dx} \right]\frac{{\partial \:^{2} w_{0} }}{{\partial \:x^{2} }} = & \sum {\:_{{j = 1}}^{{N_{D} }} } K_{j} \left( {y_{j} - w_{0} \left( {x,t} \right)} \right)\delta \:\left( {x - x_{j} } \right) \\ & + \sum {\:_{{j = 1}}^{{N_{D} }} } C_{j} \left( {\mathop {y_{j} }\limits^{.} - \frac{{\partial \:w_{0} \left( {x,t} \right)}}{{\partial \:t}}} \right)\delta \:\left( {x - x_{j} } \right) + {\mathrm{F}}({\mathrm{t}})\delta \:\left( {x - x_{a} } \right) \\ \end{aligned}$$

### Discretization and coupled equations using Galerkin method

The beam displacement is expanded in modal coordinates:

16 $$\:{w}_{0}\left(x,t\right)=\sum\:_{i=1}^{N}{q}_{i}\left(t\right){\psi\:}_{i}\left(x\right)$$

The expansion involves the beam’s eigenfunction $\:{\psi\:}_{i}\left(x\right)$where $\:N$ is the modal truncation order and $\:{q}_{i}\left(t\right)$is the generalized coordinate. For a beam with hinged supports at both ends, the th vibrational mode shape is given by^21^:

17 $$\:{\psi\:}_{i}\left(x\right)=\sqrt{\frac{2}{\rho\:AL}}\mathrm{sin}\left(\frac{i\pi\:x}{L}\right)$$

The orthonormality conditions satisfied by the vibration mode shapes of hinged-hinged beams are presented as follows:

18 $$\:\rho\:A{\int\:}_{0}^{L}{\psi\:}_{i}\left(x\right){\psi\:}_{j}\left(x\right)=\left\{\begin{array}{c}0\:\:\:\:\:\:\:\:\:\:i\ne\:j\\\:1\:\:\:\:\:\:\:\:\:\:i=j\end{array}\right.$$

Substituting Eq. (15) into Eq. (14):

19 $$\begin{gathered} \:I_{0} \sum {\:_{{i = 1}}^{N} } \psi \:_{i} \left( x \right)\ddot{q}_{i} \left( t \right) + c_{w} \sum {\:_{{i = 1}}^{N} } \psi \:_{i} \left( x \right)\dot{q}_{i} \left( t \right) + \eta \:D_{{XX}} \sum {\:_{{i = 1}}^{N} } \psi \:_{i} \prime \:\prime \:\prime \:\prime \:\left( x \right)\dot{q}_{i} \left( t \right) \hfill \\ + D_{{XX}} \sum {\:_{{i = 1}}^{N} } \psi \:_{i} \prime \:\prime \:\prime \:\prime \:\left( x \right) + \frac{{ - A_{{xx}} }}{{2L}} - \sum {\:_{{j = 1}}^{{N_{D} }} } K_{j} \left( {y_{j} - \sum {\:_{{i = 1}}^{N} } \psi \:_{i} \left( x \right)q_{i} \left( t \right)} \right)\delta \:\left( {x - x_{j} } \right) \hfill \\ - \sum {\:_{{j = 1}}^{{N_{D} }} } C_{j} \left( {\mathop {y_{j} }\limits^{.} - \sum {\:_{{i = 1}}^{N} } \psi \:_{i} \left( x \right)\dot{q}_{i} \left( t \right)} \right)\delta \:\left( {x - x_{j} } \right) = {\mathrm{F}}({\mathrm{t}})\delta \:\left( {x - x_{a} } \right) \hfill \\ \end{gathered}$$

Applying Galerkin’s method yields coupled nonlinear ODEs for the beam, TMD^21,31^:

20 $$\begin{gathered} \:I_{0} \int \: _{0}^{L} \psi \:_{m}^{2} \left( x \right)dx\ddot{q}_{m} \left( t \right) + \eta \:D_{{XX}} \int \: _{0}^{L} \psi \:_{m}^{{\prime \:\prime \:\prime \:\prime \:}} \left( x \right)\psi \:_{m} \left( x \right)dx\dot{q}_{m} \left( t \right) + D_{{XX}} \int \: _{0}^{L} \psi \:_{m}^{{\prime \:\prime \:\prime \:\prime \:}} \left( x \right)\psi \:_{m} \left( x \right)dxq_{m} \left( t \right) \hfill \\ + \sum {\:_{{j = 1}}^{{N_{D} }} } \sum {\:_{{i = 1}}^{N} } C_{j} \psi \:_{m} \left( {x_{j} } \right)\psi \:_{i} \left( {x_{j} } \right)\dot{q}_{i} \left( t \right) + \sum {\:_{{j = 1}}^{{N_{D} }} } \sum {\:_{{i = 1}}^{N} } K_{j} \psi \:_{m} \left( {x_{j} } \right)\psi \:_{i} \left( {x_{j} } \right)q_{i} \left( t \right) \hfill \\ + \sum {\:_{{ijk}} } \frac{{ - A_{{xx}} }}{{2L}}\int \: _{0}^{L} \psi \:_{i}^{{\prime \:}} \left( x \right)\psi \:_{j}^{{\prime \:}} \left( x \right)dx\int \: _{0}^{L} \psi \:_{k}^{{\prime \:\prime \:}} \left( x \right)\psi \:_{m} \left( x \right)dxq_{i} \left( t \right)q_{j} \left( t \right)q_{k} \left( t \right) - \sum {\:_{{j = 1}}^{{N_{D} }} } C_{j} \psi \:_{m} \left( {x_{j} } \right)\dot{y}_{j} \hfill \\ - \sum {\:_{{j = 1}}^{{N_{D} }} } K_{j} \psi \:_{m} \left( {x_{j} } \right)y_{j} = {\mathrm{F}}\left( {\mathrm{t}} \right)\psi \:_{m} \left( x \right)\left( {m = {\mathrm{1,2}}, \ldots \:..N} \right) \hfill \\ \end{gathered}$$

In adherence to D’Alembert’s principle, the governing motion equations for the TMD are established.

$$\:{{M}_{j}{\ddot{y}}_{j}+C}_{j}{\dot{y}}_{j}+{K}_{j}{y}_{j}-\sum\:_{i=1}^{N}{C}_{j}{\psi\:}_{i}\left({x}_{i}\right){\dot{q}}_{i}\left(t\right)-\sum\:_{i=1}^{N}{K}_{j}{\psi\:}_{i}\left({x}_{i}\right){q}_{i}\left(t\right)=0\left(j=\mathrm{1,2},\dots\:..{N}_{D}\right)\:\:\:\:\:\:\:\:\:\:\:\:\:\left(21\right)$$

## Results

In this investigation the external transverse excitation, represented by $\:F\left(t\right)$, is characterized as an impulsive force localized at a constant position ($\:x={x}_{a}$​) along the beam’s length.

22 $$\:F\left(t\right)=\:\:\:\left\{\begin{array}{c}{F}_{e}{sin}(2\pi\:t/T)\:\:\:\:\:0\le\:t\le\:T/2\\\:0\:\:\:\:\:\:\:\:\:\:\:\:\:\:\:\:\:\:t\ge\:T/2\end{array}\right.$$

### Validation

To validate our numerical model’s accuracy, simulation results were meticulously compared with established findings in the existing literature, specifically from Sheng et al.^21^. For a direct and valid comparison, the numerical beam model was configured with identical characteristics (geometry, material properties, and boundary conditions) as those reported in the validating study. This analysis considers a linear beam with the following properties:

$$\:L=1\:m,\:{x}_{1}=0.5\:m,\:{x}_{a}=0.3\:m,\:{F}_{e}=10\:N,\:T=\left(0.04/\pi\:\right)s,\:EI=1\:N.{m}^{2},\rho\:A=1\frac{Kg}{m},\:{M}_{1}=0.1\:kg,\:{c}_{w}=0$$

**Fig. 2:**
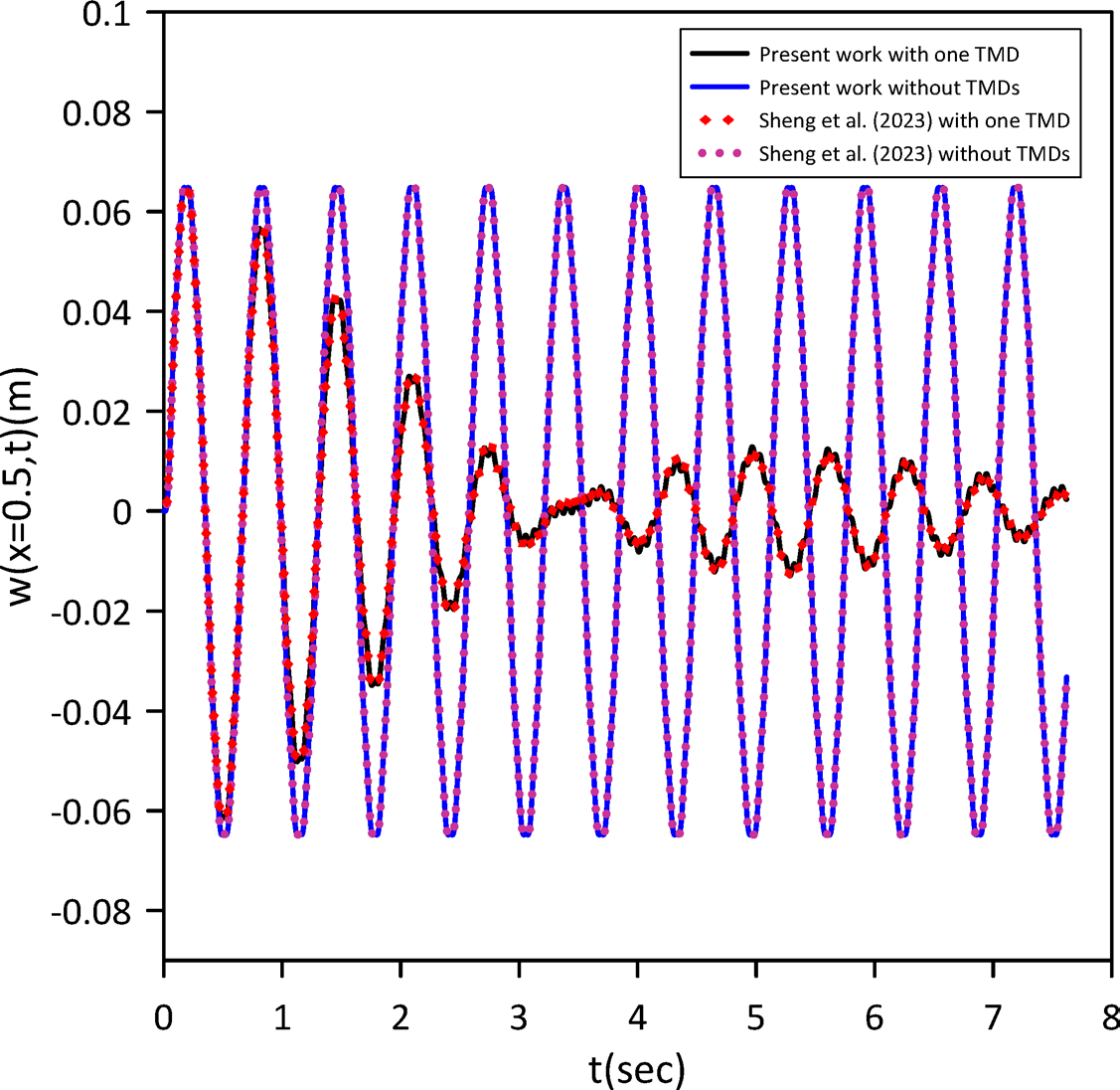
Transverse midspan deflection over time without TMDs and with one tuned mass damper at the midpoint neglecting internal damping.

**Fig. 3:**
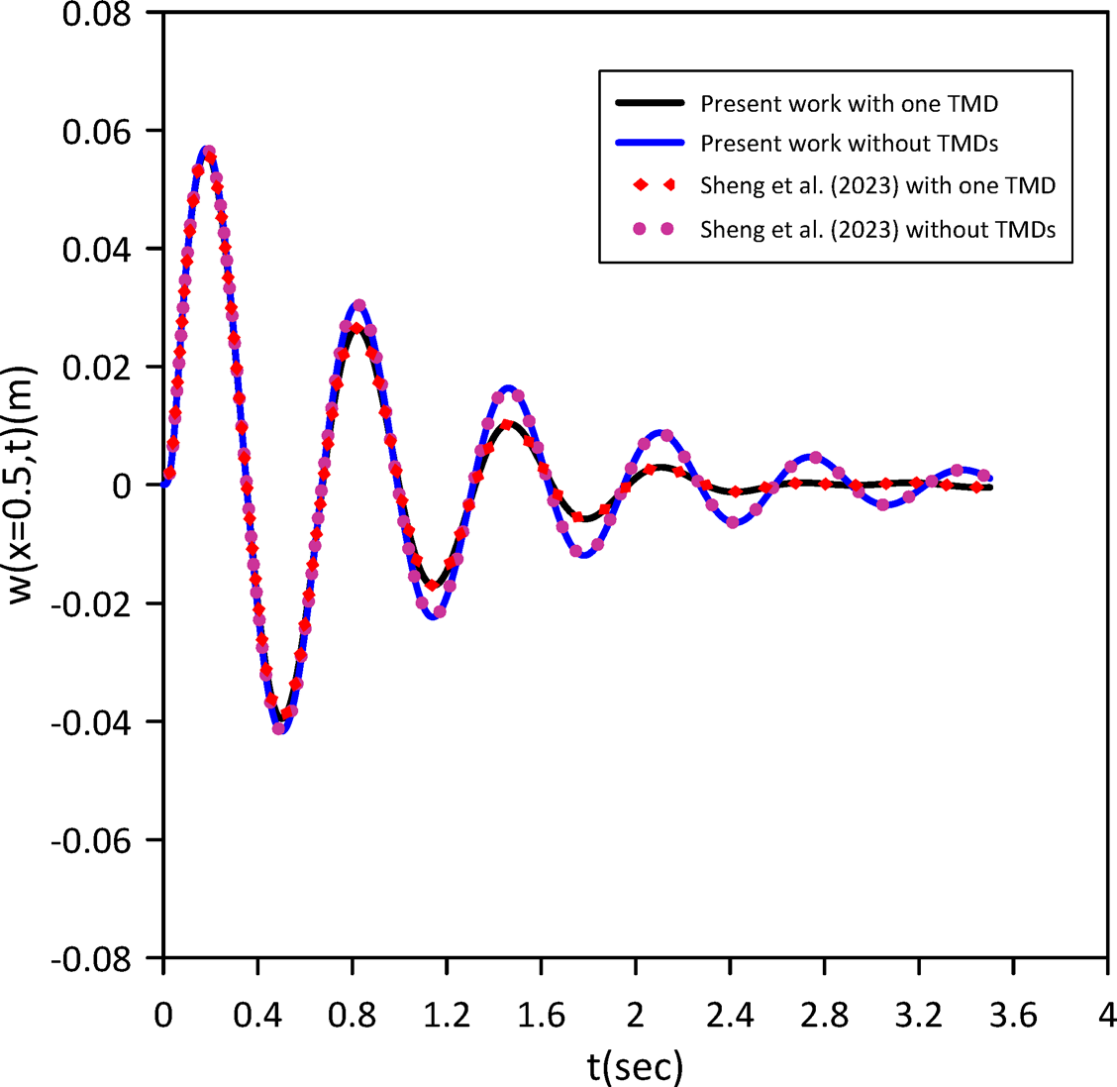
Transverse midspan deflection over time without TMDs and with one tuned mass damper at the midpoint considering internal damping.

As demonstrated in the Figs. 2 and 3, our results show strong agreement with the validating study, confirming the accuracy of our model.

### Linear beam

To understand the dynamic behavior of the beam, we start with a linear analysis, initially disregarding any nonlinearity. For this analysis, we’re looking at a beam with length *L* = 1 m, a cross-section area *A* = 1.2788×$\:{10}^{-4}{\:\:m}^{2}$, a moment of inertia $\:I=4.8356\times\:{10}^{-12}$ $\:{\:\:m}^{4}$, a modulus of elasticity *E*=206.8×$\:{10}^{9}\:\mathrm{G}\mathrm{P}\mathrm{a}$, density$\:\rho\:=7820\:$Kg/m^3^ an internal damping coefficient $\:\eta\:=0.02,\:$impulsive force amplitude $\:{F}_{e}=10\:N$, impulsive force position$\:{x}_{a}=0.3$ m, period $\:\:T=0.02\:$s, a mass of the tuned mass damper $\:{M}_{i}=0.01\:kg$ and a motion damping coefficient $\:{c}_{w}=0$.

A modal convergence study was performed to ensure the accuracy of the dynamic response. The solution was computed iteratively with an increasing number of modes. The results for maximum displacement was found to change by less than 1% upon including the fifth mode, indicating sufficient convergence. Consequently, the first five modes were deemed adequate and employed for all further analyses.

#### Without internal damping

**Fig. 4:**
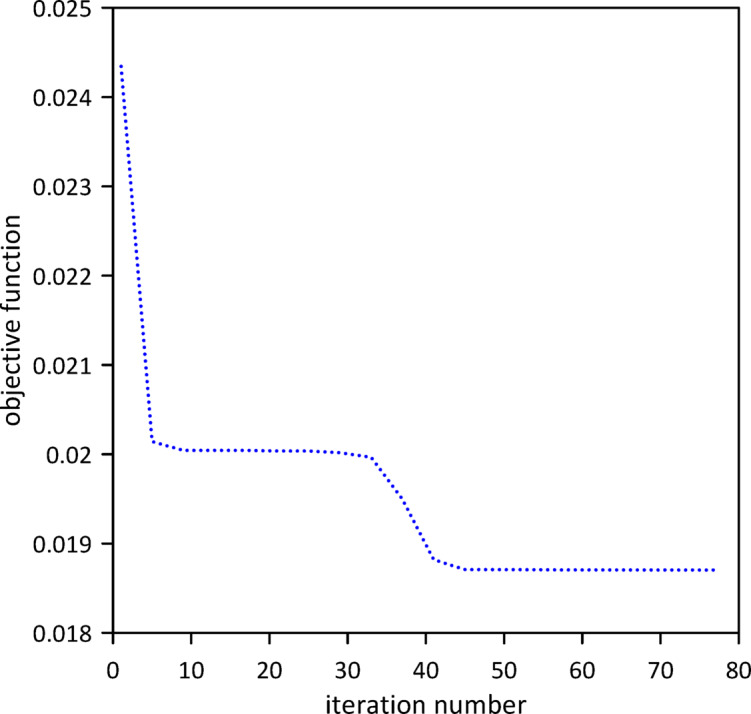
Objective function convergence for a linear beam with one TMD neglecting internal damping.

**Fig. 5:**
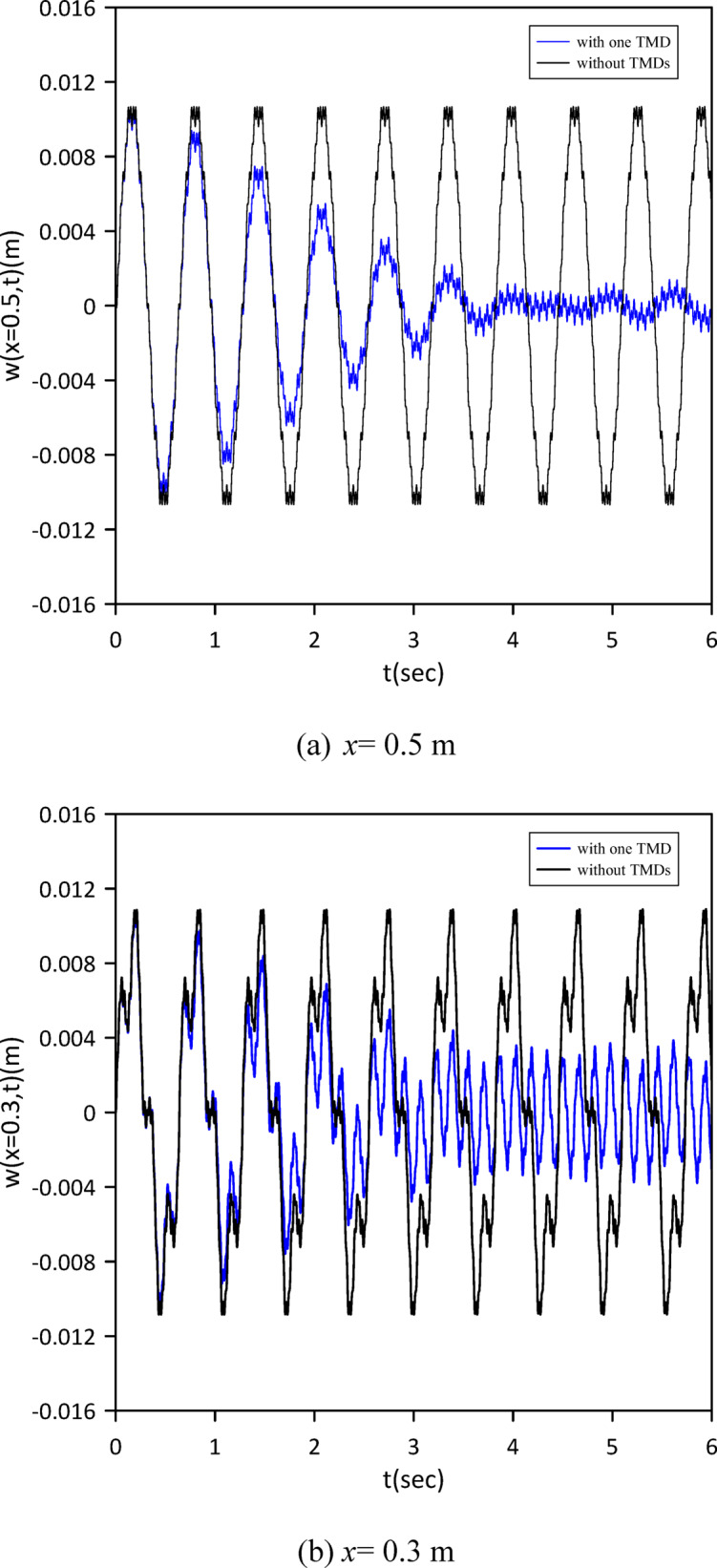
Linear beam: comparison of cases without and with one TMD (neglecting internal damping).

In this section, we neglect internal damping within the beam. To evaluate the effectiveness of vibration control, we first analyzed the response of the linear beam before the implementation of any tuned mass dampers (TMDs). Subsequently, a single TMD was attached, and its position, stiffness, and damping coefficient were optimized using Particle Swarm Optimization (PSO) to achieve the most favorable damping characteristics as shown in Fig. 4. The area under the beam’s response curve served as the objective function its mathematical description is given by Eq. (14). The PSO algorithm converged to optimal TMD parameters: $\:{x}_{1}=0.5041\:m$, $\:{k}_{1}=0.9371$N/m, $\:{c}_{1}=0.0176$ N.s/m. The effectiveness of the optimized single tuned mass damper (TMD) in mitigating vibrations is clearly demonstrated in Fig. 5, which shows the significant decrease in the beam’s response.

23 $$\:A\left(x\right)={\int\:}_{{t}_{1}}^{{t}_{2}}w\left(x,t\right)dt$$

Where $\:{t}_{1}$ and $\:{t}_{1}$ denote the initial and final time points over which the area is calculated.

**Fig. 6:**
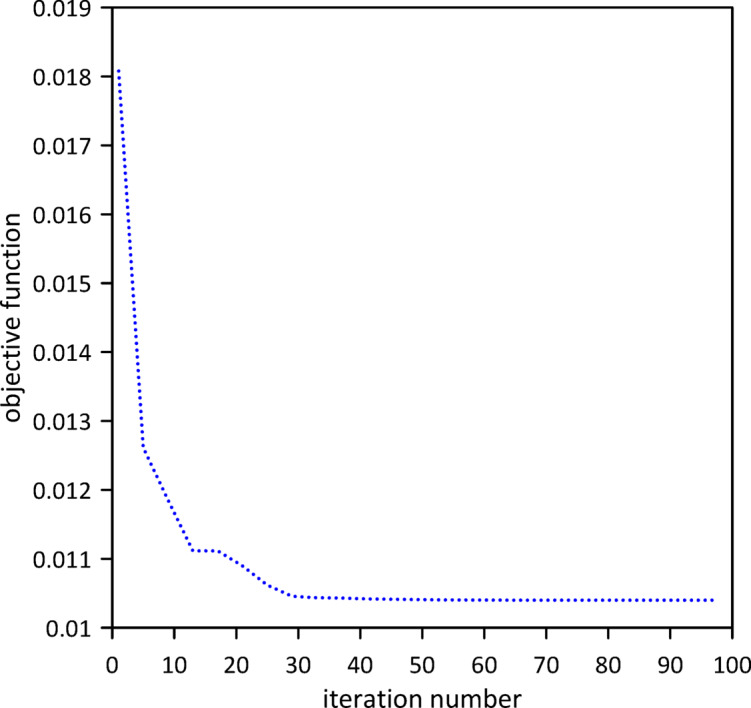
Objective function convergence for a linear beam with two TMDs neglecting internal damping.

**Fig. 7:**
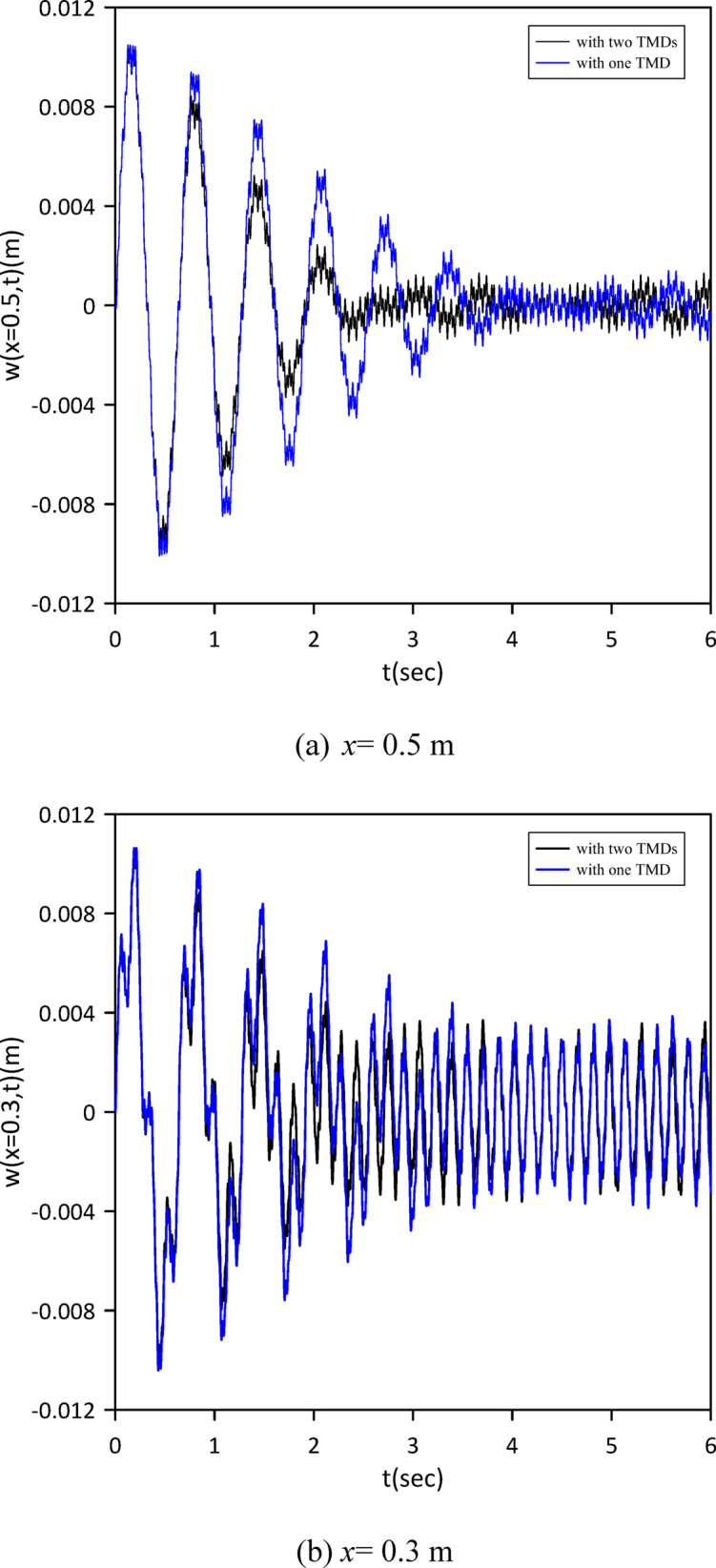
Linear beam deflection: comparison of cases with one and two TMDs (neglecting internal damping).

The investigation was extended to include a second tuned mass damper (TMD). In this subsequent analysis, the system now comprises two TMDs attached to the beam, with the objective remaining to minimize the area under the beam’s response curve. Similar to the single TMD case, Particle Swarm Optimization (PSO) was again employed as shown in Fig. 6 to determine the optimal positions, stiffnesses, and damping coefficients for both TMDs simultaneously. This multi-TMD configuration aims to further enhance vibration suppression. Optimal TMD parameters were found to be: $\:{x}_{1}=0.5074\:m$, $\:{k}_{1}=1.04372$N/m, $\:{c}_{1}=0.01645$ N.s/m, $\:{x}_{2}=0.6\:m$, $\:{k}_{2}=0.80625$N/m, $\:{c}_{2}=0.0116$ N.s/m. As depicted in Fig. 7, the optimized two-TMD system proves highly effective in reducing vibrations, showcasing a significant attenuation in the beam’s response when compared to the single TMD configuration.

**Fig. 8:**
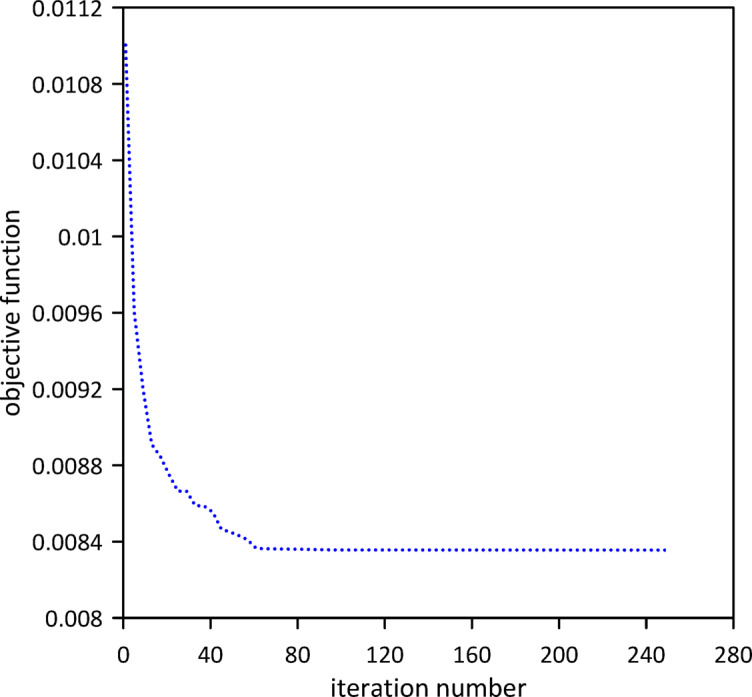
Objective function convergence for a linear beam with three TMDs neglecting internal damping.

**Fig. 9:**
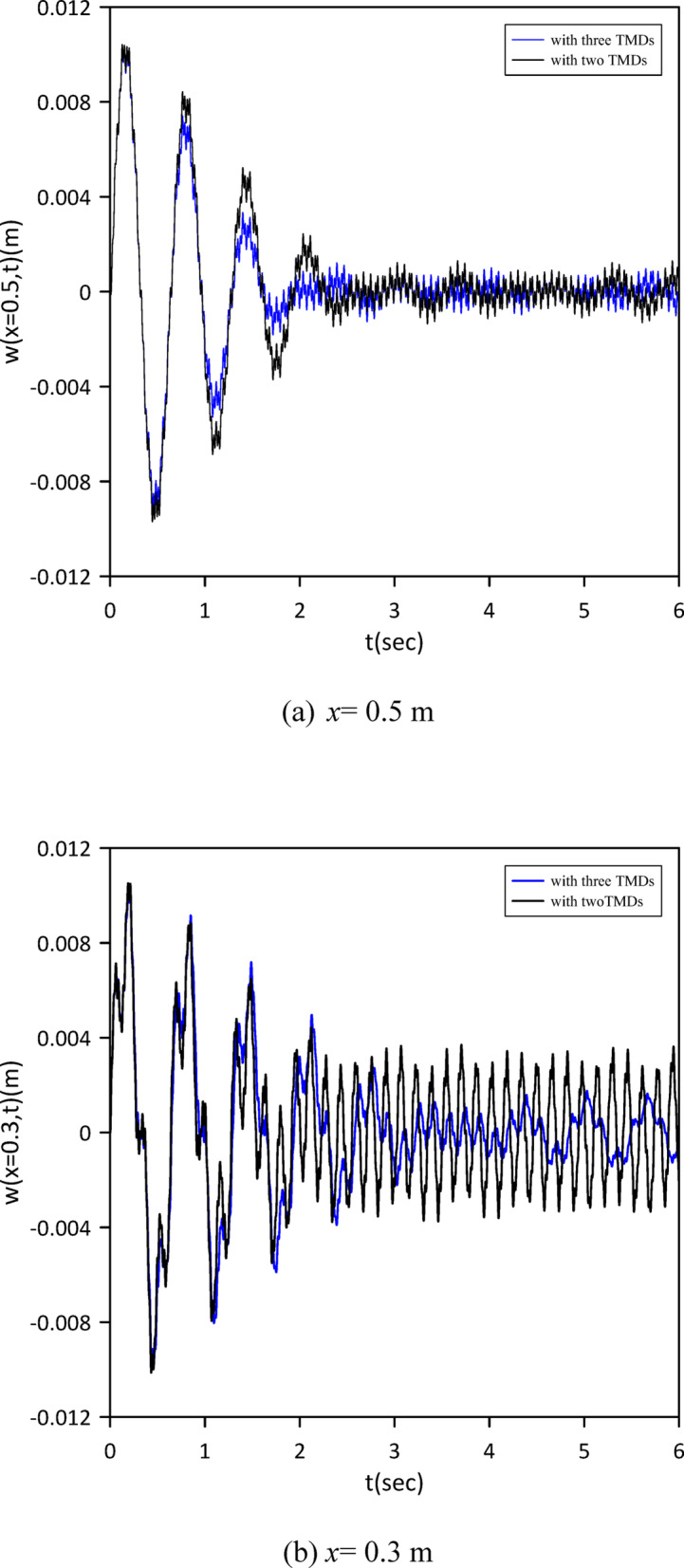
Linear beam deflection: comparison of cases with two and three TMDs (neglecting internal damping).

To further enhance vibration suppression, the investigation was extended to include a third tuned mass damper (TMD), resulting in a system comprising three TMDs attached to the beam. As shown in Fig. 8, Particle Swarm Optimization (PSO) was again employed to simultaneously determine the optimal positions, stiffnesses, and damping coefficients for all three TMDs. Optimal TMD parameters were found to be: $\:{x}_{1}=0.5025\:m$, $\:{k}_{1}=1.1437\:$N/m, $\:{c}_{1}=0.017$ N.s/m, $\:{x}_{2}=0.5\:m$, $\:{k}_{2}=0.8961\:\mathrm{N}$/m, $\:{c}_{2}=0.01239$ N.s/m, $\:{x}_{3}=0.4977\:m$, $\:{k}_{3}=0.7059\:\mathrm{N}$/m, $\:{c}_{3}=0.0098$ N.s/m. Figure 9 illustrates that the optimized three-TMD system leads to an additional decrease in vibration compared to the two-TMD setup, exhibiting attenuation in the beam’s response.

The vibration suppression performance of the linear beam without internal damping is summarized in Table 1. A consistent improvement is observed with each additional TMD. The three-TMD configuration achieves the best results, with an 89.7% reduction in maximum midspan response after 3 s and a settling time of 12 s.it is a dramatic improvement over the uncontrolled system, which fails to settle within 5%.

**Table 1:** Quantitative assessment of vibration suppression performance for the linear beam (without internal damping) across single and multiple TMD configurations.

Performance Metric	Without TMDs	With one TMD	With two TMDs	With three TMDs
Maximum midspan response after 3 s (m)	0.0107	0.0029	0.0014	0.0011
Reduction (%)	reference	72.897	86.9	89.7
Settling Time (5%) (s)	infinite	24.5	23	12

For a single TMD, the optimal position is exactly at the midpoint, the first-mode antinode. This placement maximizes the relative motion between the TMD and the beam, ensuring efficient energy transfer from the dominant vibration mode. With two TMDs, the optimizer places one TMD very close to the midpoint and the other at a location around $\:0.6$ m. The second TMD, while still interacting with the first mode, also provides some influence on the second mode, reflecting a compromise to reduce contributions from higher-modal components excited by the impulsive load. When a third TMD is added, all three positions cluster tightly around the midpoint. The optimizer simply adds mass and damping near the same critical location rather than distributing absorbers to higher-mode antinodes. Consequently, the performance gain from the third TMD is marginal, as seen in Table 1.

#### With internal damping

**Fig. 10:**
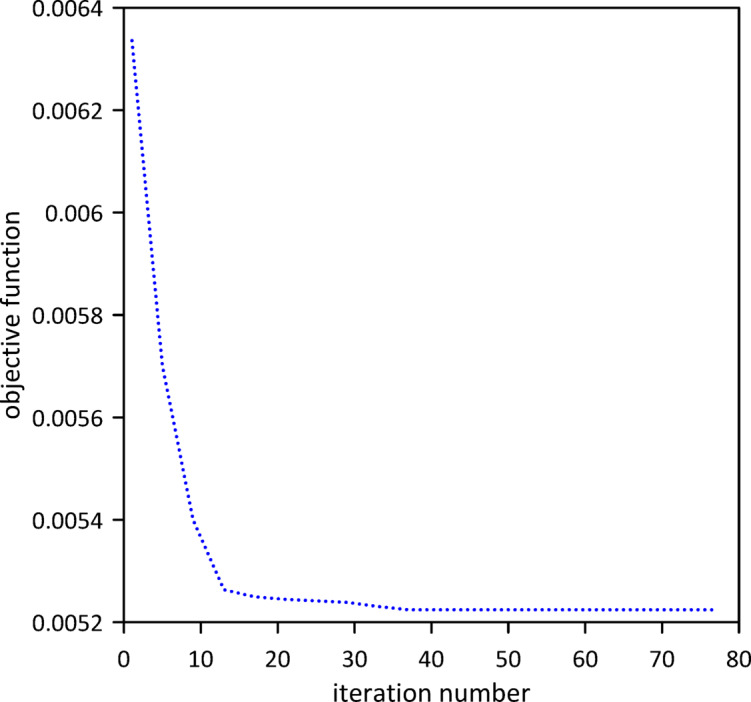
Objective function convergence for a linear beam with one TMD with internal damping.

**Fig. 11:**
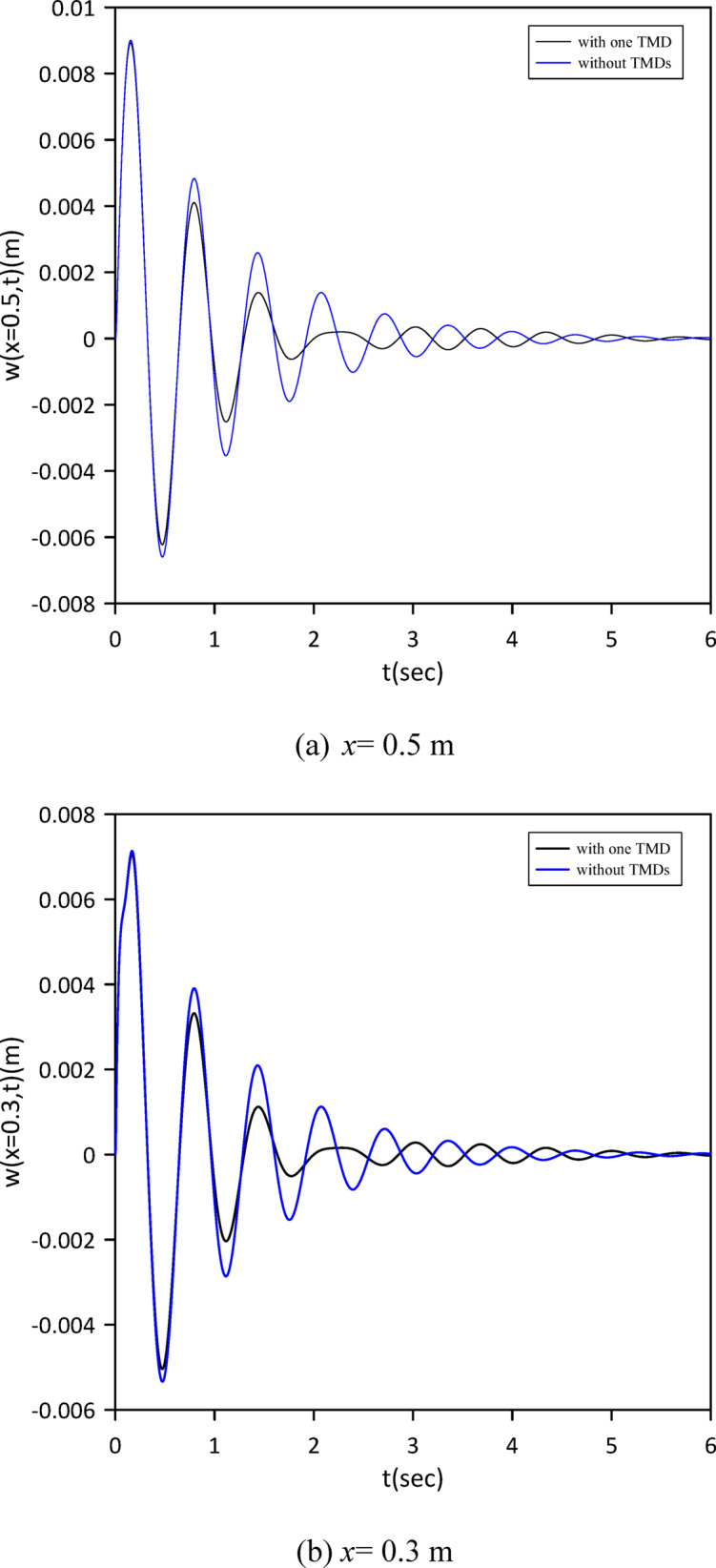
Linear beam deflection: comparison of cases without and with one TMD (with internal damping). (b) x = 0.3 m.

This section advances the model by considering the beam’s internal damping, a departure from the previous analysis. We initially characterized the linear beam’s response without any tuned mass dampers (TMDs) to set a new baseline. Subsequently, a single TMD was attached, and its optimal position, stiffness, and damping coefficient were determined via Particle Swarm Optimization (PSO) as shown in Fig. 10 to achieve the best damping performance given the presence of internal damping. The optimal TMD parameters resulting from the PSO algorithm were: $\:{x}_{1}=0.4999\:m$, $\:{k}_{1}=0.8873\:N$/m, $\:{c}_{1}=0.0232$ N.s/m. Figure 11 clearly demonstrates the effectiveness of the optimized single tuned mass damper (TMD) in mitigating vibrations, illustrating a significant decrease in the beam’s response.

**Fig. 12:**
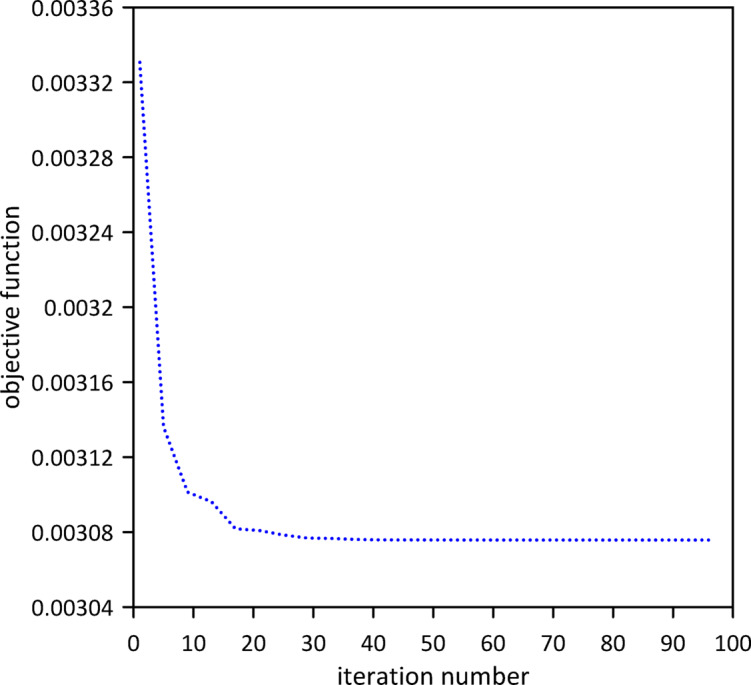
Objective function convergence for a linear beam with two TMDs with internal damping.

**Fig. 13:**
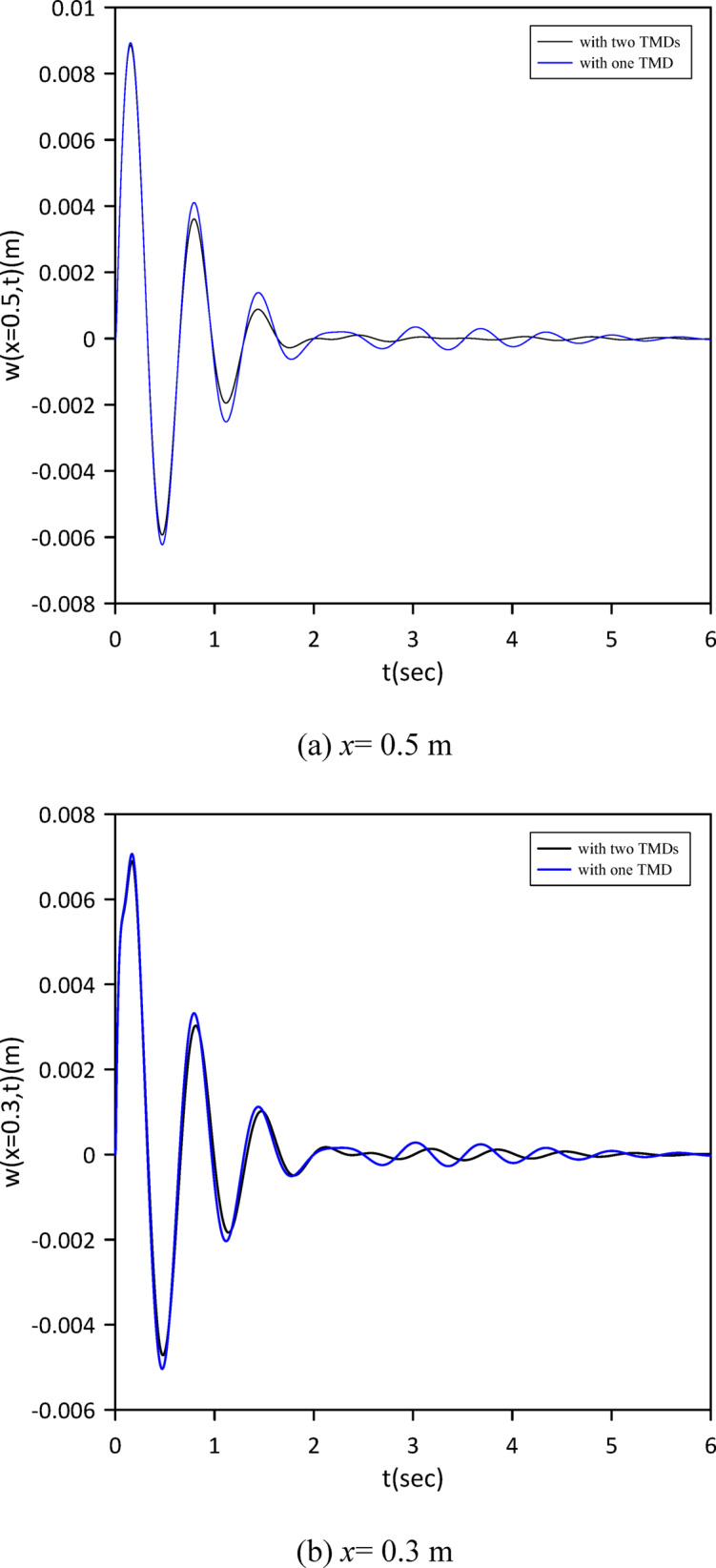
Linear beam deflection: comparison of cases with one and two TMDs (with internal damping). (a) x = 0.5 m, (b) x = 0.3 m.

The investigation was extended to a two-TMD system attached to the beam. As shown in Fig. 12, Particle Swarm Optimization (PSO) was again employed to simultaneously determine the optimal positions, stiffnesses, and damping coefficients for both TMDs, aiming to further enhance vibration suppression. Optimal TMD parameters were found to be: $\:{x}_{1}=0.4966\:m$, $\:{k}_{1}=0.7272\:\mathrm{N}$/m, $\:{c}_{1}=0.019$ N.s/m, $\:{x}_{2}=0.5031\:m$, $\:{k}_{2}=1.0017\:\mathrm{N}$/m, $\:{c}_{2}=0.0251$ N.s/m. Figure 13 demonstrates the optimized two-TMD system’s effectiveness in mitigating vibrations, showing attenuation in the beam’s response compared to the single TMD configuration.

**Fig. 14:**
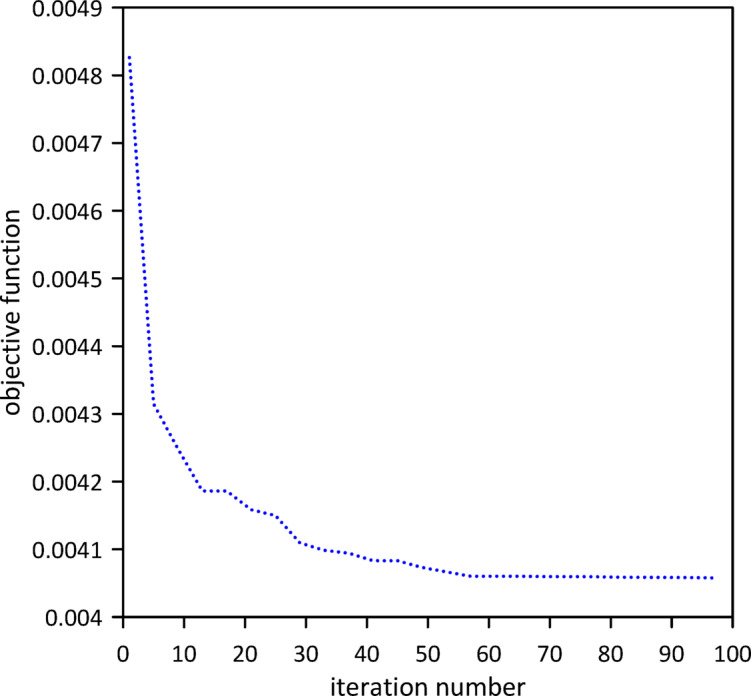
Objective function convergence for a linear beam with three TMDs with internal damping.

**Fig. 15:**
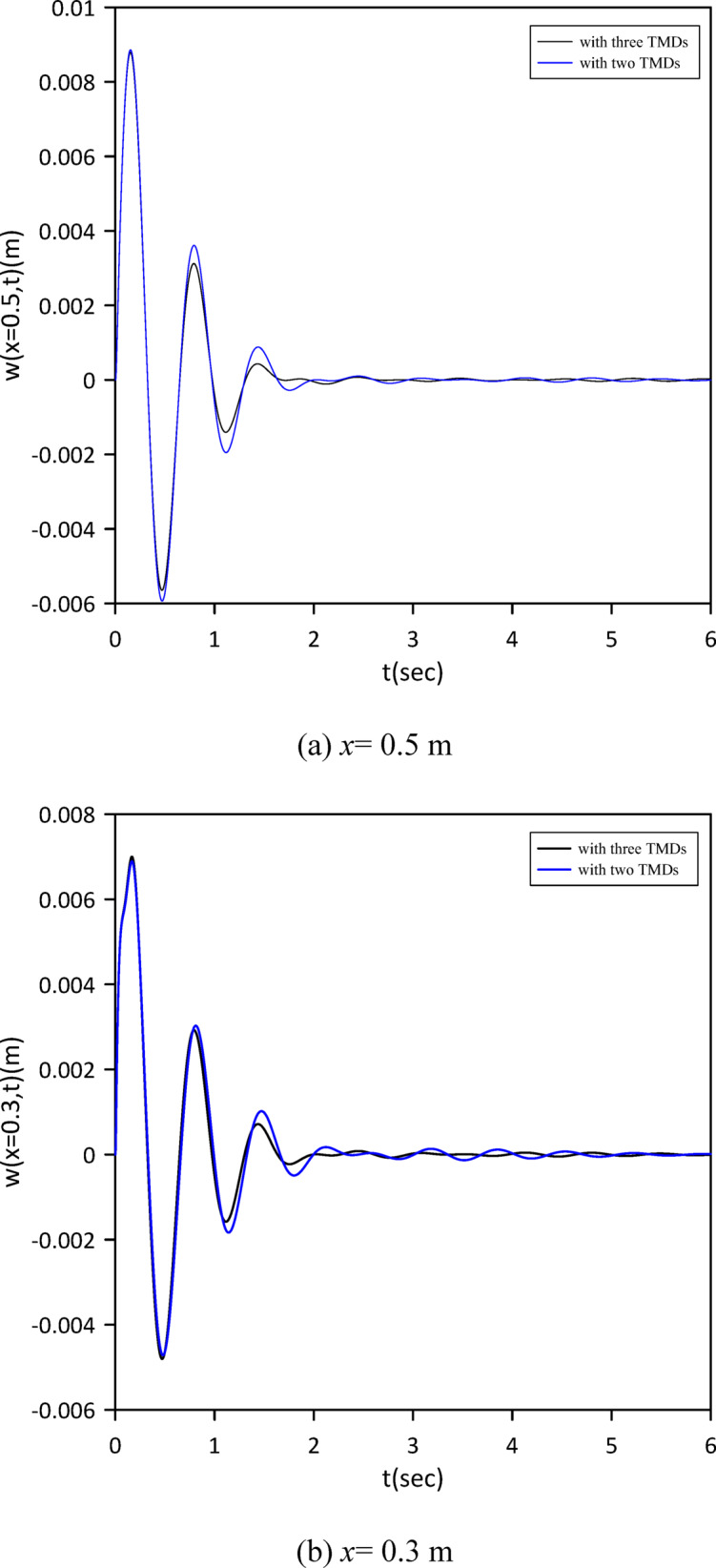
Linear beam deflection: comparison of cases with two and three TMDs (with internal damping). (a) x = 0.5 m, (b) x = 0.3 m.

The investigation expanded to a three-TMD system attached to the beam, aiming for enhanced vibration suppression. As shown in Fig. 14, Particle Swarm Optimization (PSO) was again employed to simultaneously determine the optimal positions, stiffnesses, and damping coefficients for all three TMDs. Optimal TMD parameters were found to be: $\:{x}_{1}=0.4855\:m$, $\:{k}_{1}=0.6168\:\mathrm{N}$/m, $\:{c}_{1}=0.0158$ N.s/m, $\:{x}_{2}=0.4916\:m$, $\:{k}_{2}=1.0888\:\mathrm{N}$/m, $\:{c}_{2}=0.0258$ N.s/m, $\:{x}_{3}=0.5003\:m$, $\:{k}_{3}=0.8186\:$N/m, $\:{c}_{3}=0.0192$ N.s/m. Compared to the two-TMD setup, Fig. 15 demonstrates that the optimized three-TMD system achieves a further reduction in vibration, resulting in attenuated beam response.

**Table 2:** Quantitative assessment of vibration suppression performance for the linear beam (with internal damping) across single and multiple TMD configurations.

Performance Metric	Without TMDs	With one TMD	With two TMDs	With three TMDs
Maximum midspan response after 3 s (×10⁻⁴ m)	5.45	3.48	0.594	0.44
Reduction (%)	reference	36.15	89.1	91.93
Settling Time (5%) (s)	3.1	1.86	1.55	1.26

Table 2 presents a quantitative assessment of the vibration suppression performance for the linear beam with internal damping. The results demonstrate that increasing the number of TMDs yields substantial and continuous improvements across all metrics. The three-TMD configuration proves most effective, reducing the maximum midspan response after 3 s from $\:5.45\times\:{10}^{-4}$ m to $\:4.40\times\:{10}^{-5}$ m, a 91.93% reduction. The 5% settling time also improves progressively, decreasing from 3.10 s in the uncontrolled case to 1.26 s with three TMDs. Notably, while the two-TMD configuration already achieves an 89.1% reduction, the addition of a third absorber provides further, albeit diminishing, gains. This suggests that even in a linear system with inherent damping, there exists an optimal range beyond which additional absorbers yield progressively smaller returns.

With internal damping present, the behaviour changes. The single TMD again sits at the midpoint. For two TMDs, both optimal positions converge very close to the midpoint. The internal damping already attenuates higher modes, so the optimizer concentrates both absorbers at the location of maximum first-mode response, leaving no need to target the second mode. Adding a third TMD results in positions that also cluster near the midpoint, and the incremental improvement is small (Table 2). The inherent damping, combined with the already effective two-TMD configuration, leaves little room for further enhancement.

### Nonlinear beam

This section delves into the nonlinear dynamic behavior of the beam, aiming to provide a more comprehensive analysis of its response to forced excitation. The underlying physical parameters of the beam are kept consistent with the linear model, but its nonlinear constitutive relationships are now activated.

A modal convergence study ensured the accuracy of the dynamic response. The solution was computed iteratively with an increasing number of modes until the maximum displacement changed by less than 1% upon including the fifth mode. This indicated convergence, and thus the first five modes were used for all subsequent analyses.

#### Without internal damping

**Fig. 16:**
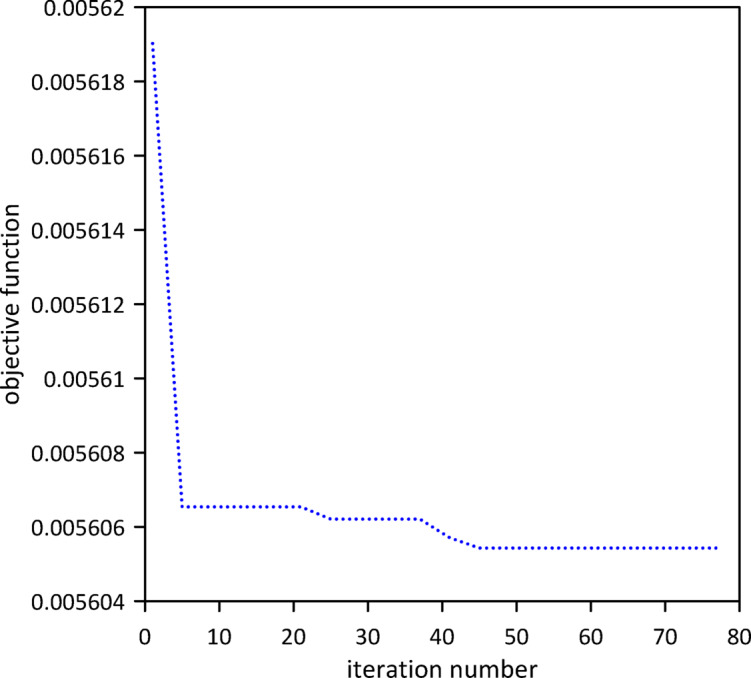
Objective function convergence for a nonlinear beam with one TMD neglecting internal damping.

**Fig. 17:**
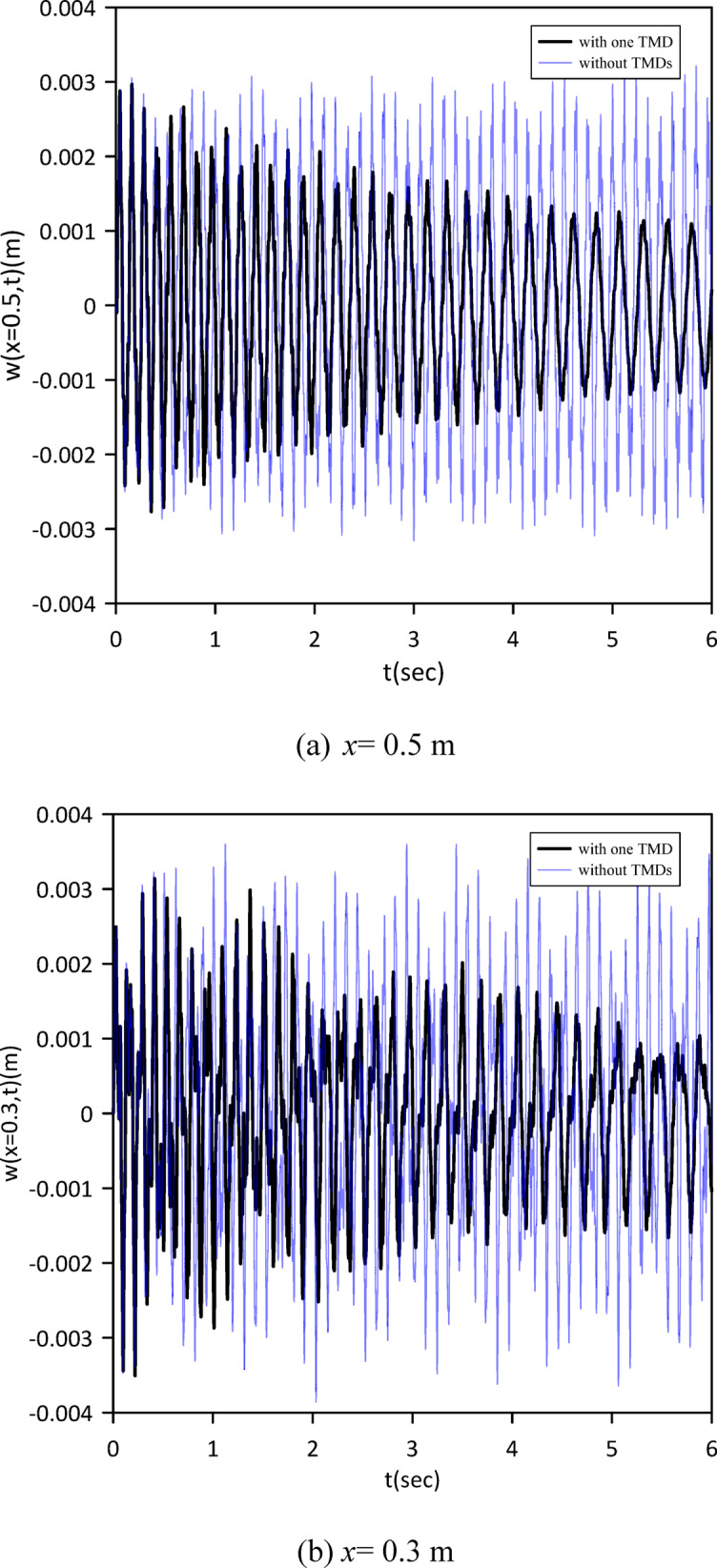
Nonlinear beam deflection: comparison of cases without and with one TMD (neglecting internal damping). (b) x = 0.3 m.

In this section, we analyze the nonlinear beam’s dynamic behavior, neglecting internal damping. To evaluate vibration control effectiveness, we first characterized the nonlinear beam’s response without any tuned mass dampers (TMDs). Subsequently, a single TMD was attached, and its optimal position, stiffness, and damping coefficient were determined using Particle Swarm Optimization (PSO), as shown in Fig. 16. The PSO algorithm converged to optimal TMD parameters: $\:{x}_{1}=0.507\:m$, $\:{k}_{1}=2.0967\:N$/m, $\:{c}_{1}=0.4055$ N.s/m. Figure 17 clearly demonstrates the effectiveness of the optimized single tuned mass damper (TMD) in mitigating vibrations, illustrating a significant decrease in the beam’s response.

**Fig. 18:**
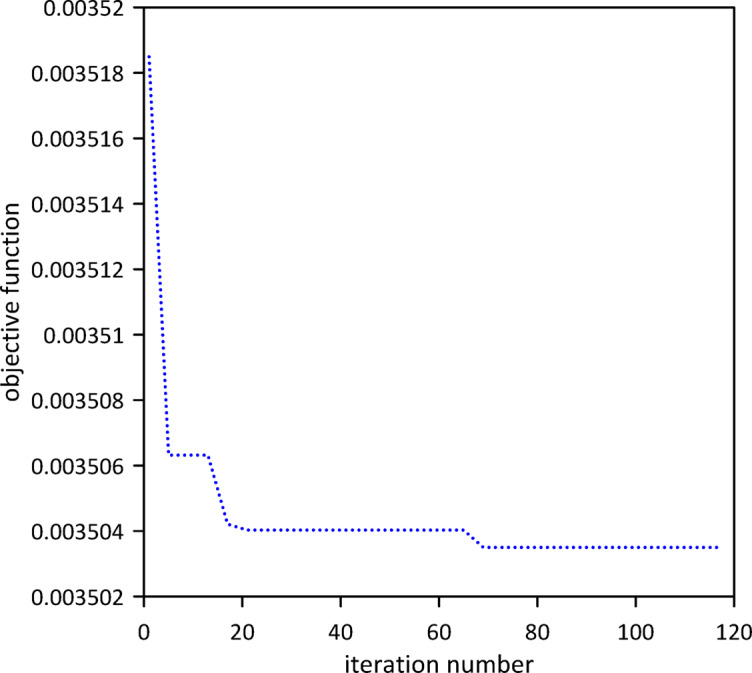
Objective function convergence for a nonlinear beam with two TMDs neglecting internal damping.

**Fig. 19:**
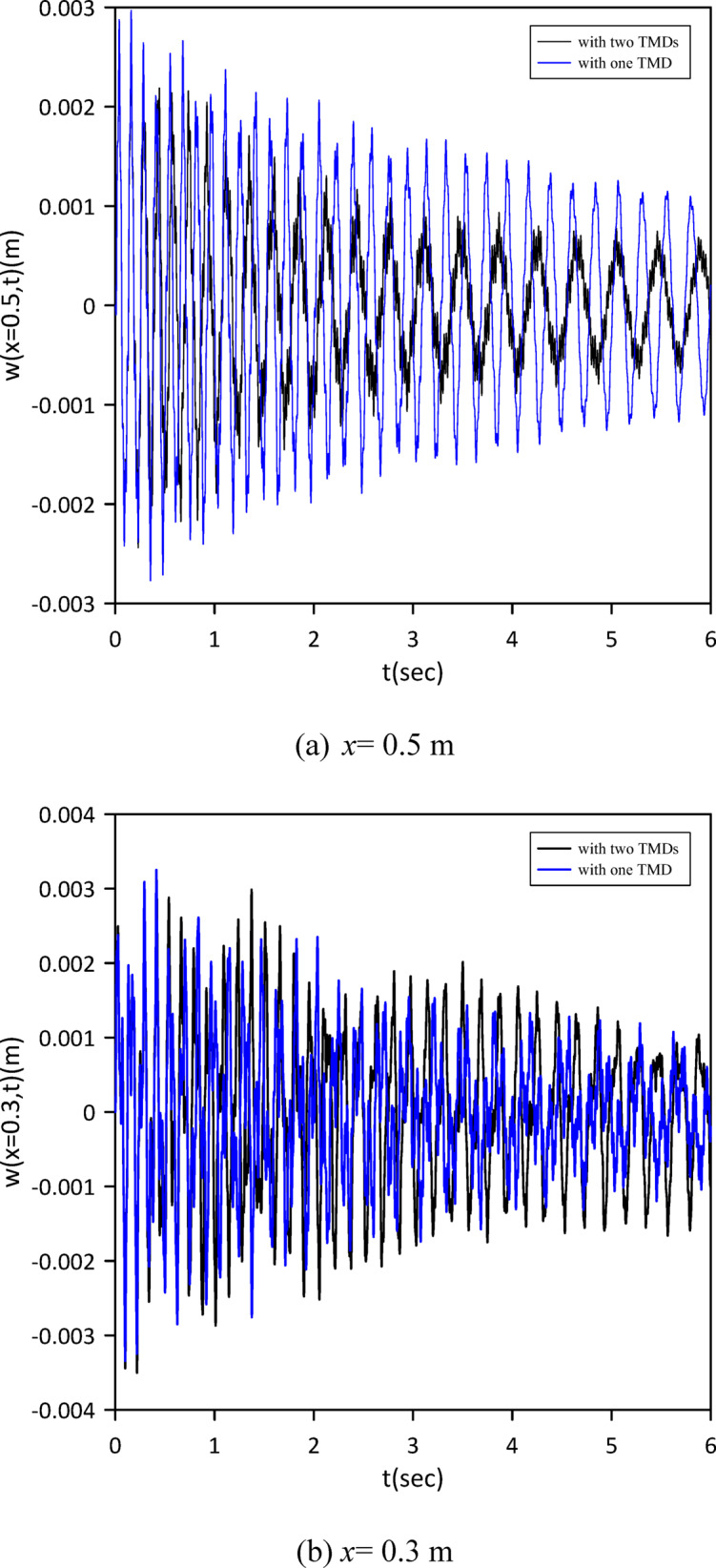
Nonlinear beam deflection: comparison of cases with one and two TMDs (neglecting internal damping). (b) x = 0.3 m.

The investigation was extended to a two-TMD system attached to the beam. As Fig. 18 shows, Particle Swarm Optimization (PSO) was again used to simultaneously determine the optimal positions, stiffnesses, and damping coefficients for both TMDs, aiming to further enhance vibration suppression. Optimal TMD parameters were:$\:{x}_{1}=0.5072\:m$, $\:{k}_{1}=12.2\:\mathrm{N}$/m, $\:{c}_{1}=0.1774$ N.s/m, $\:{x}_{2}=0.5105\:m$, $\:{k}_{2}=4.3391\:\mathrm{N}$/m, $\:{c}_{2}=0.102$ N.s/m. Figure 19 demonstrates the optimized two-TMD system’s effectiveness in mitigating vibrations, showing attenuation in the beam’s response compared to the single TMD configuration.

**Fig. 20:**
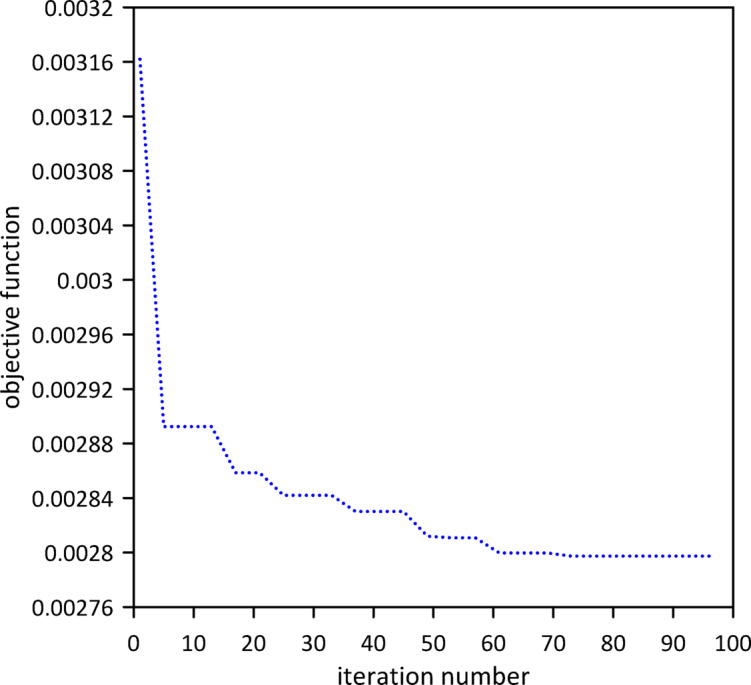
Objective function convergence for a nonlinear beam with three TMDs neglecting internal damping.

**Fig. 21:**
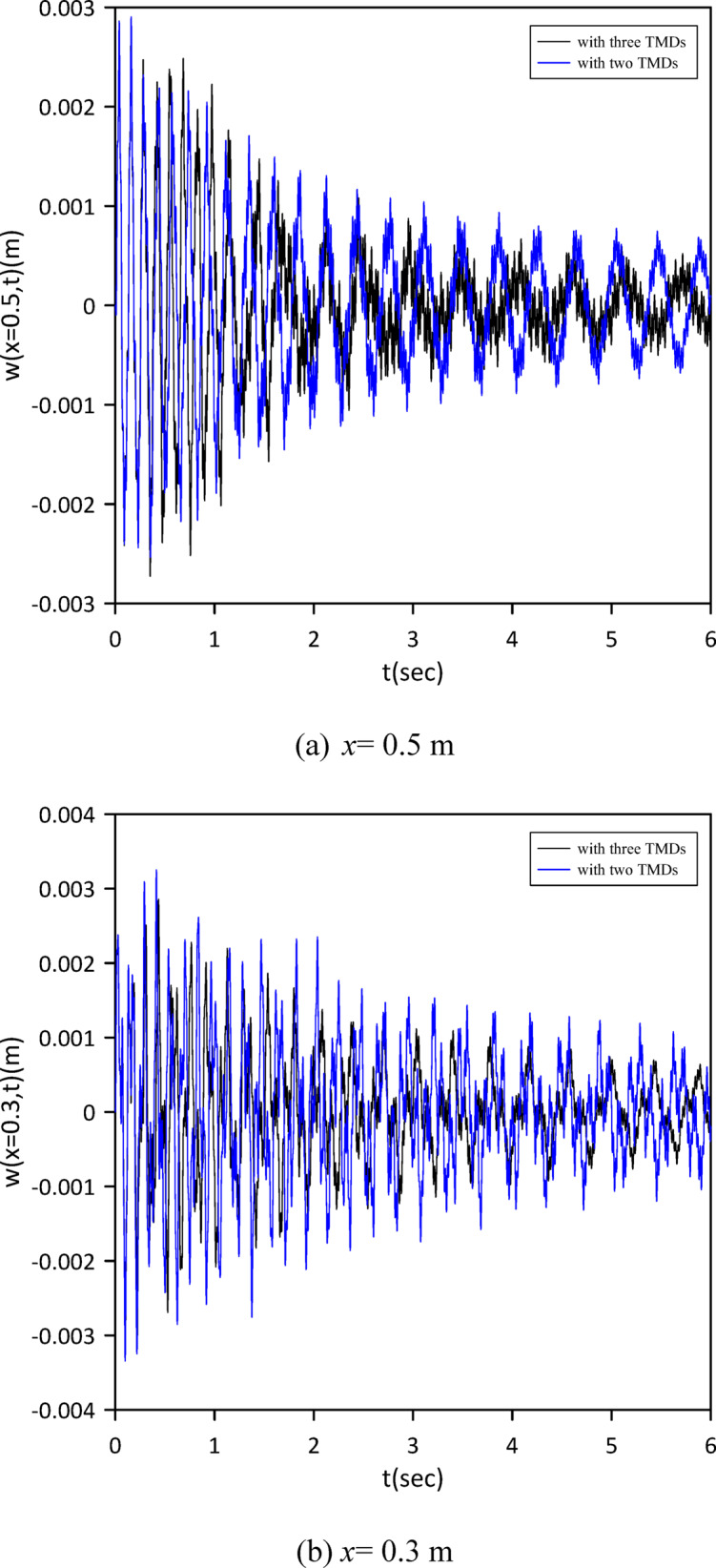
Nonlinear beam deflection: comparison of cases with two and three TMDs (neglecting internal damping).

Building on previous configurations, the study progressed to analyze a three-TMD system attached to the beam. As illustrated in Fig. 20, Particle Swarm Optimization (PSO) was utilized to simultaneously ascertain the optimal positions, stiffnesses, and damping coefficients for all three devices, aiming for superior vibration suppression. The resulting optimal TMD parameters were: $\:{x}_{1}=0.5050\:m$, $\:{k}_{1}=3.5407\:$N/m, $\:{c}_{1}=0.2002$ N.s/m, $\:{x}_{2}=0.5038\:m$, $\:{k}_{2}=5.2856\:$N/m, $\:{c}_{2}=0.0083$ N.s/m, $\:{x}_{3}=0.5243\:m$, $\:{k}_{3}=14.0792\:\mathrm{N}$/m, $\:{c}_{3}=0.0070$ N.s/m. Figure 21 demonstrates that the optimized three-TMD system achieves a further reduction in vibration compared to the two-TMD setup, resulting in an attenuated beam response.

The vibration suppression performance of the nonlinear beam without internal damping is summarized in Table 3. A consistent improvement is observed with each additional TMD. The three-TMD configuration achieves the best results, with a 76.25% reduction in maximum midspan response after 3 s and a settling time of 12 s which is a substantial improvement over the uncontrolled system.

**Table 3:** Quantitative assessment of vibration suppression performance for the nonlinear beam (without internal damping) across single and multiple TMD configurations.

Performance Metric	Without TMDs	With one TMD	With two TMDs	With three TMDs
Maximum midspan response after 3 s (m)	0.0032	0.0017	0.001	0.00076
Reduction (%)	reference	46.88	68.75	76.25
Settling Time (5%) (s)	infinite	20	15	12

For a single TMD, the optimum remains near the midpoint. With two TMDs, both absorbers cluster within the first-mode lobe, still centred around the midpoint. The optimizer does not place a TMD near the second-mode antinode; instead, it keeps both devices where the dominant nonlinear response is largest.

A third TMD again clusters near the same region, and the performance improvement over the two-TMD case is modest (Fig. 21). The nonlinearity does not alter the fundamental trend: additional absorbers are added near the location of maximum modal displacement, yielding diminishing returns.

#### With internal damping

**Fig. 22:**
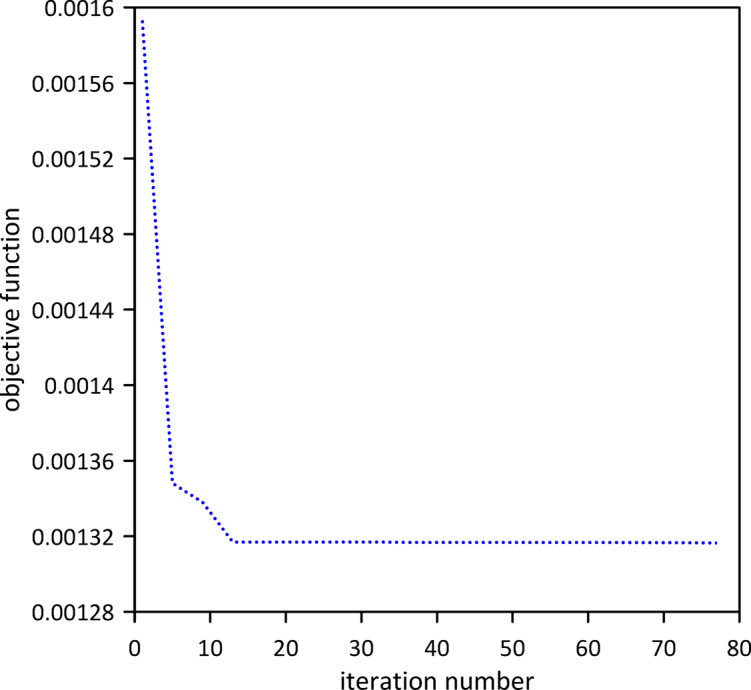
Objective function convergence for a nonlinear beam with one TMD with internal damping.

**Fig. 23:**
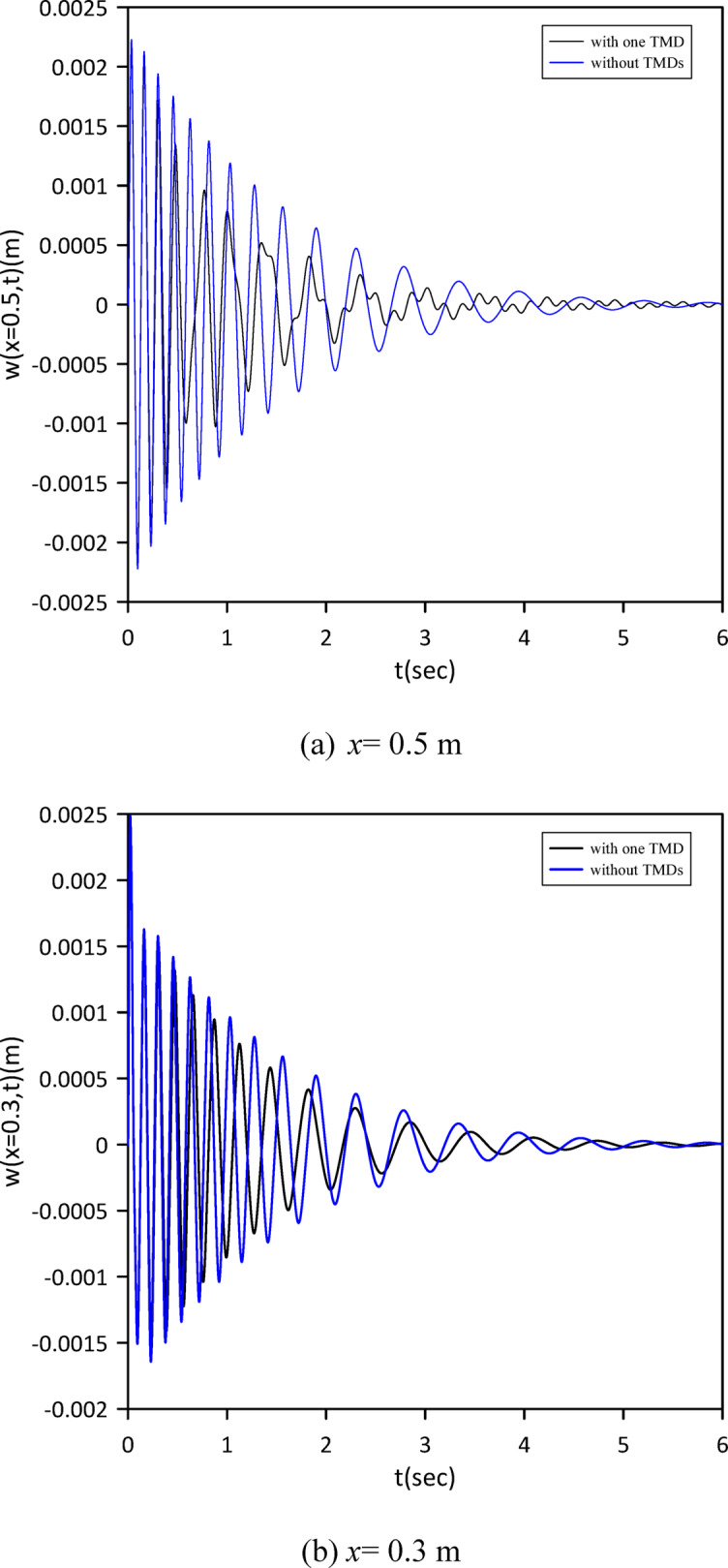
Nonlinear beam deflection: comparison of cases without and with one TMD (with internal damping). (b) x = 0.3 m.

**Fig. 24:**
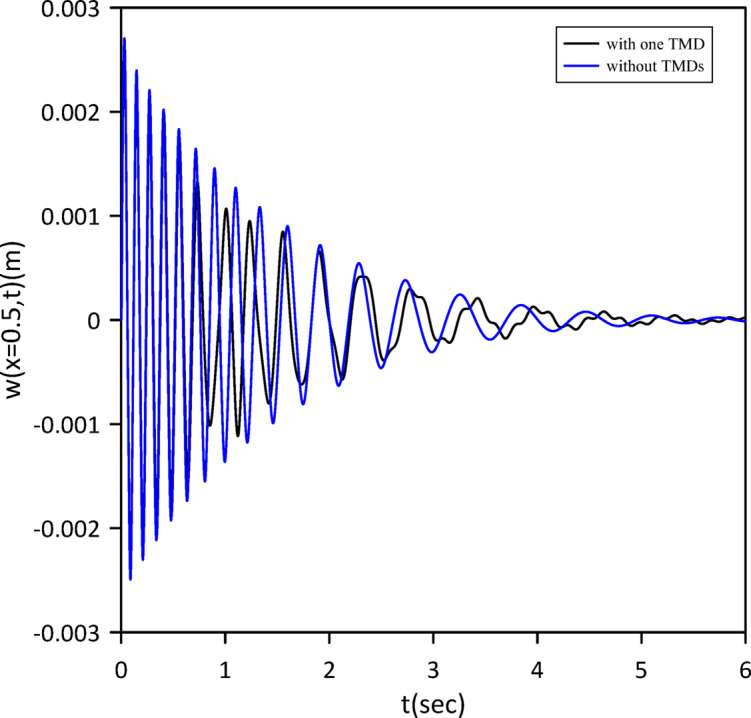
Nonlinear beam midspan deflection(excitation at *x* = 0.5): comparison of cases without and with one TMD (with internal damping).

Building on prior analysis, this section refines the model by incorporating the beam’s internal damping. We established a new baseline by characterizing the nonlinear beam’s response without TMDs. Subsequently, a single TMD’s optimal position, stiffness, and damping coefficient were determined via Particle Swarm Optimization (PSO) (Fig. 22) to maximize damping performance given the internal damping. Optimal TMD parameters were: $\:{x}_{1}=0.4884\:m$, $\:{k}_{1}=13.1314\:N$/m, $\:{c}_{1}=0.0081$ N.s/m. Figure 23 clearly demonstrates the effectiveness of the optimized single tuned mass damper (TMD) in mitigating vibrations. The effectiveness of the TMD was further verified by changing the location of the impulsive force to *x* = 0.5 m. The results, presented in Fig. 24, confirm a reduction in vibration achieved with a single TMD.

**Fig. 25:**
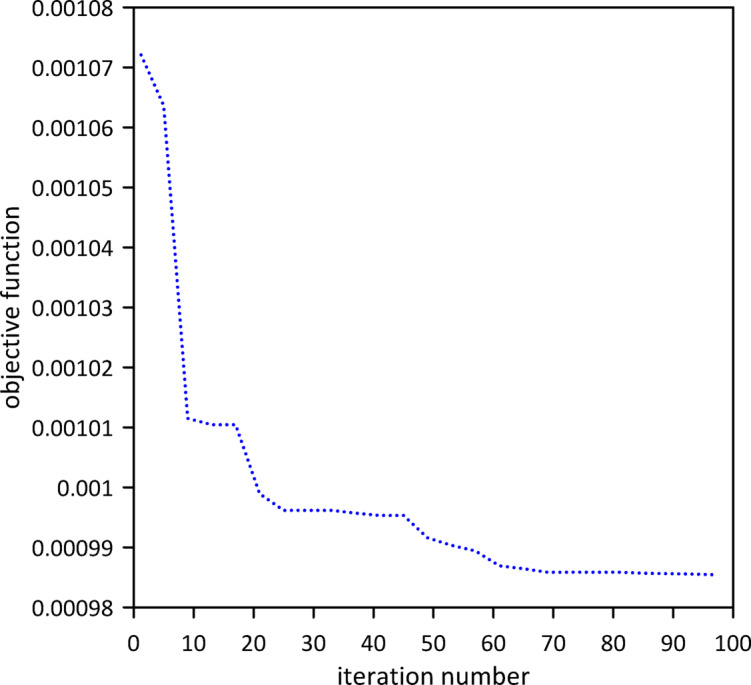
Objective function convergence for a nonlinear beam with two TMDs with internal damping.

**Fig. 26:**
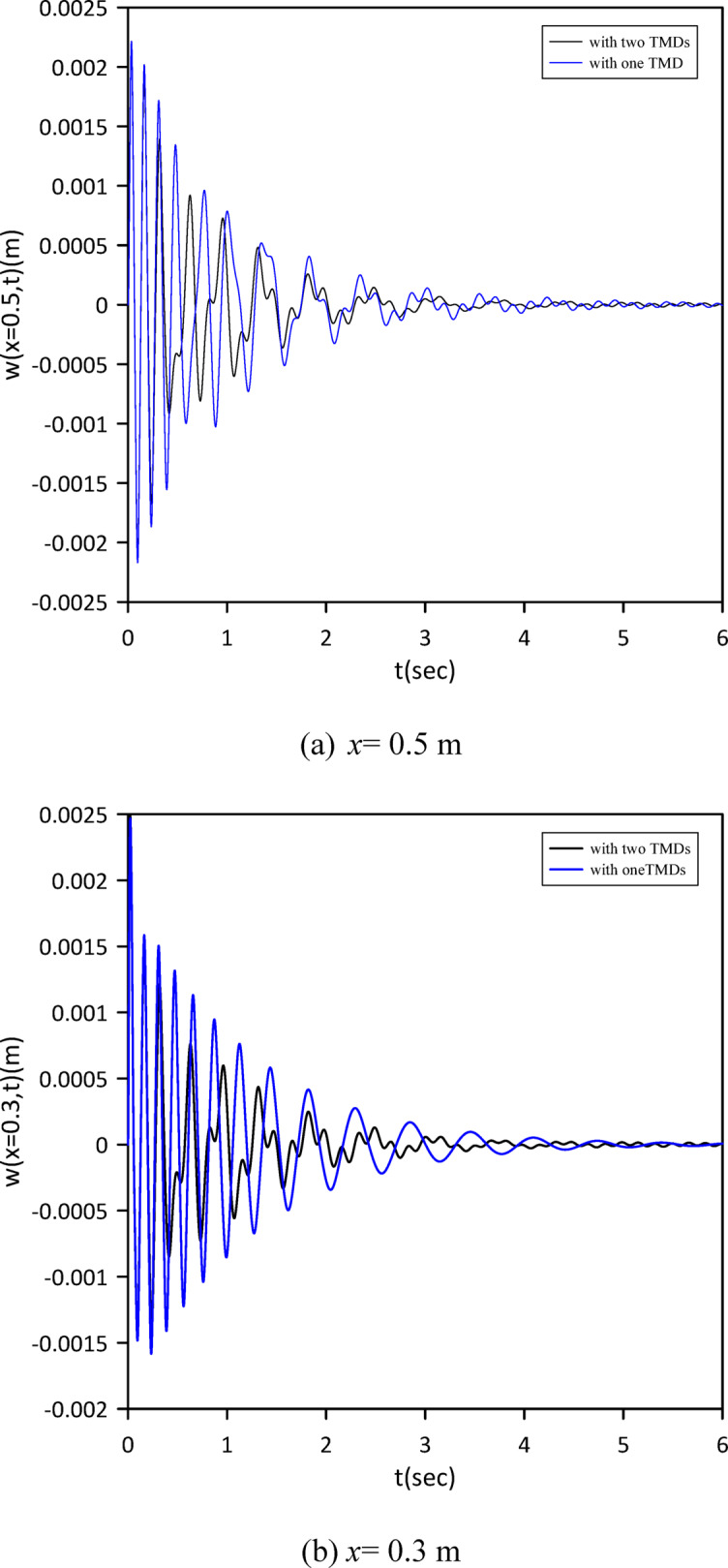
Nonlinear beam deflection: comparison of cases with one and two TMDs (with internal damping). (b) x = 0.3 m.

**Fig. 27:**
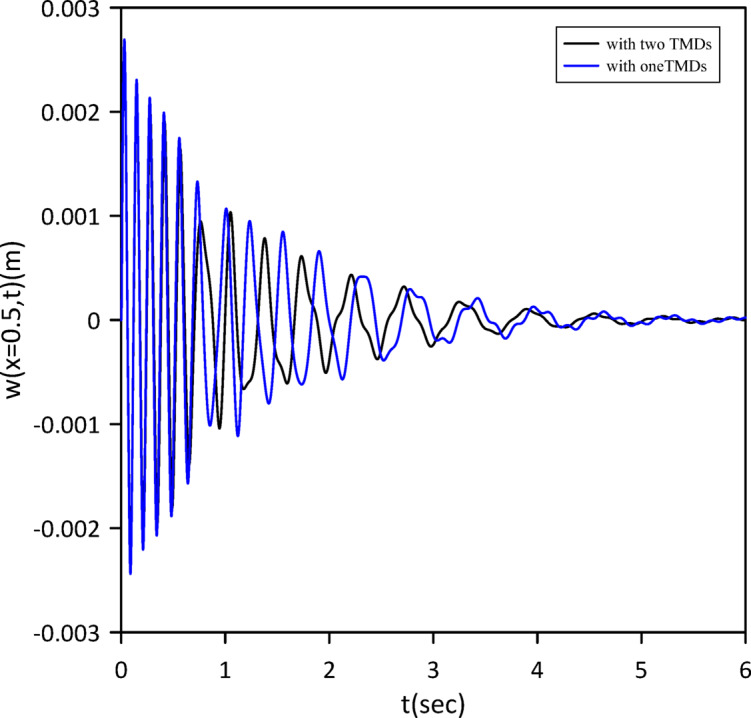
Nonlinear beam midspan deflection(excitation at *x* = 0.5): comparison of cases with one and two TMDs (with internal damping).

The investigation was extended to a two-TMD system attached to the beam. As Fig. 25 shows, Particle Swarm Optimization (PSO) was again used to simultaneously determine the optimal positions, stiffnesses, and damping coefficients for both TMDs, aiming to further enhance vibration suppression. Optimal TMD parameters were:$\:{x}_{1}=0.5292\:m$, $\:{k}_{1}=136654\:\mathrm{N}$/m, $\:{c}_{1}=0.0124$ N.s/m, $\:{x}_{2}=0.5082\:m$, $\:{k}_{2}=13.1052\:\mathrm{N}$/m, $\:{c}_{2}=0.0067$ N.s/m. As illustrated in Fig. 26, the optimized two-TMD system provides a slight, yet noticeable, attenuation in the beam’s response when compared to the single TMD configuration. As shown in Fig. 27, the application of two TMDs successfully decreased the vibration amplitude when the impulsive force was relocated to *x* = 0.5 m.

**Fig. 28:**
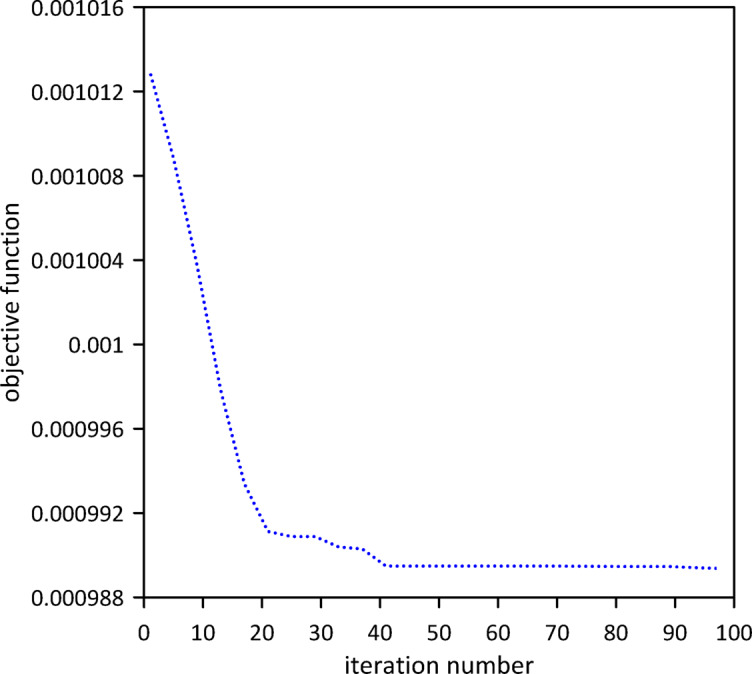
Objective function convergence for a nonlinear beam with three TMDs with internal damping.

**Fig. 29:**
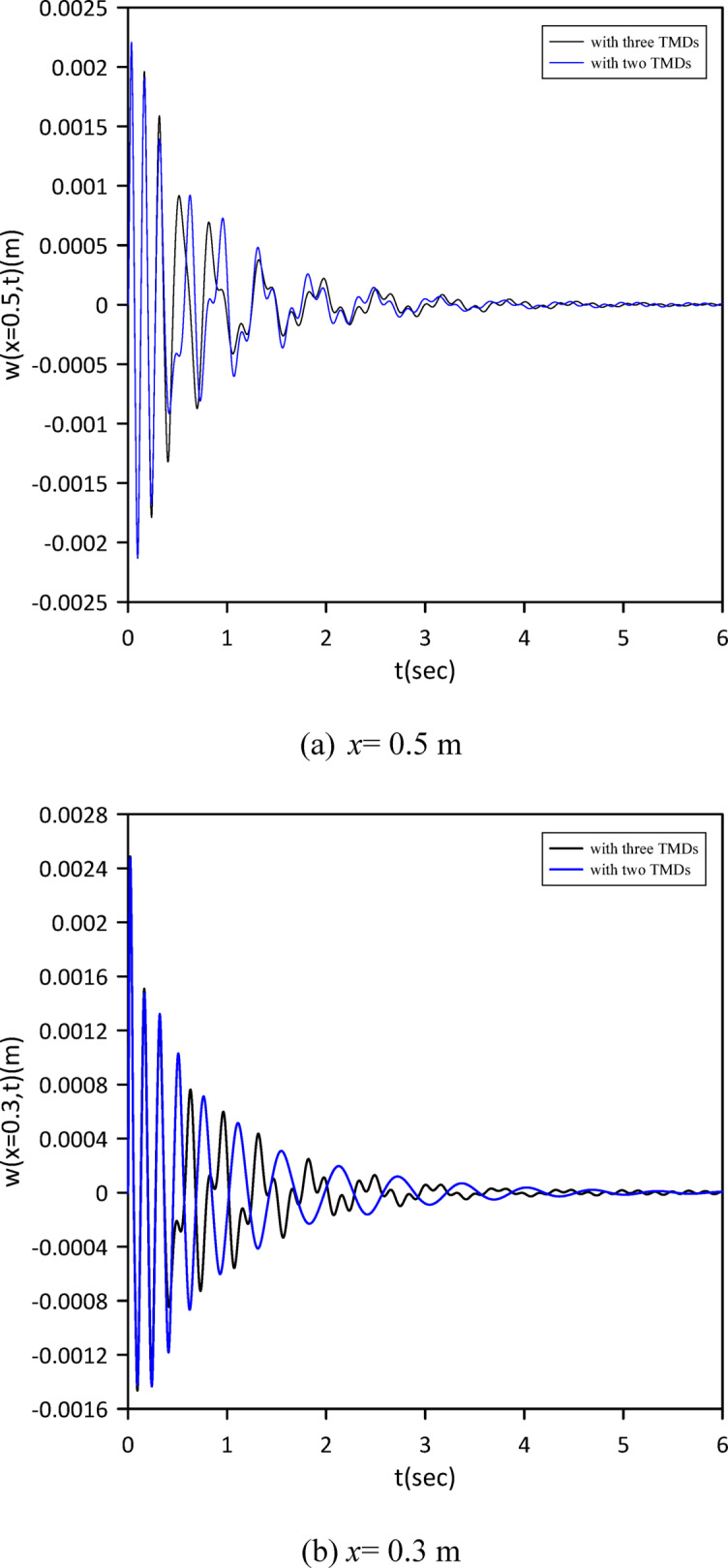
Nonlinear beam deflection: comparison of cases with two and three TMDs (with internal damping). (b) x = 0.3 m.

**Fig. 30:**
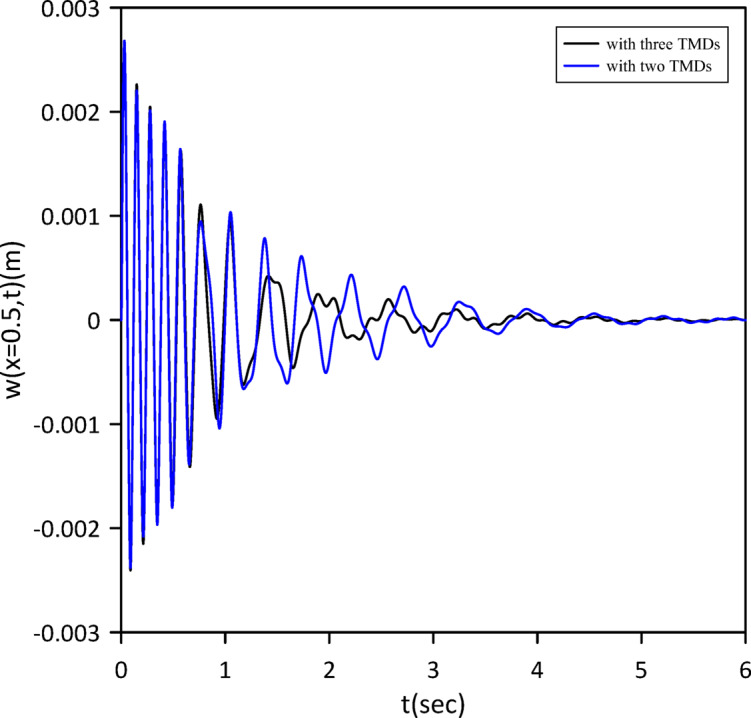
Nonlinear beam midspan deflection(excitation at *x* = 0.5): comparison of cases with two and three TMDs (with internal damping).

The study progressed to analyze a three-TMD system attached to the beam, aiming for superior vibration suppression. As Fig. 28 illustrates, Particle Swarm Optimization (PSO) was utilized. The resulting optimal TMD parameters were $\:{x}_{1}=0.4915\:m$, $\:{k}_{1}=4.3121\:$N/m, $\:{c}_{1}=0.0672$ N.s/m, $\:{x}_{2}=0.5118\:m$, $\:{k}_{2}=4.3668\:$N/m, $\:{c}_{2}=0.0216$ N.s/m, $\:{x}_{3}=0.4748\:m$, $\:{k}_{3}=13.4986\:\mathrm{N}$/m, $\:{c}_{3}=0.0112$ N.s/m. Compared to the two-TMD setup, Fig. 29 indicates that the optimized three-TMD system does not yield better attenuation in the beam’s vibration response so it is better to use only two tuned mass dampers. With the impulsive force applied at *x* = 0.5 m, the three-TMD configuration demonstrated its effectiveness by achieving a reduction in vibration, as shown in Fig. 30.

**Table 4:** Quantitative assessment of vibration suppression performance for the nonlinear beam (with internal damping) across single and multiple TMD configurations.

Performance Metric	Without TMDs	With one TMD	With two TMDs	With three TMDs
Maximum midspan response after 3 s (×10⁻⁴ m)	2.526	1.4	0.693	0.85
Reduction (%)	Reference	44.58	72.57	66.35
Settling Time (5%) (s)	3.96	3.68	2.51	2.52

Table 4 presents a quantitative assessment of the vibration suppression performance across the evaluated TMD configurations. The results demonstrate that even a single optimized TMD yields a substantial improvement, reducing the maximum midspan response after 3 s by 44.58%, from $\:2.5264\times\:{10}^{-4}$ m to $\:1.4\times\:{10}^{-4}$ m. The two-TMD configuration proved to be the most effective, achieving a 72.57% reduction and improving the 5% settling time from 3.96 s to 2.51 s. Notably, increasing the number of absorbers to three resulted in diminishing returns; the reduction efficiency dropped to 66.35%, and the maximum midspan response after 3 s increased slightly to $\:8.5\times\:{10}^{-5}$ m. This suggests that for this nonlinear system, the introduction of a third absorber induces modal interference rather than providing additional benefit. These findings underscore the necessity of optimizing the number of absorbers to identify the most efficient structural configuration.

The single TMD is again near the midpoint. For two TMDs, one remains at the midpoint while the other is slightly offset—both still within the first-mode lobe. Importantly, adding a third TMD does not improve performance; in fact, the reduction efficiency drops (Table 4). The optimal three-TMD positions are also clustered near the midpoint, but the combination of nonlinearity and internal damping appears to cause detrimental modal interactions when too many absorbers are crowded into the same spatial region. This explains why the two-TMD configuration is optimal for the nonlinear damped beam.

## Conclusion

This study comprehensively investigated the optimal design of Tuned Mass Dampers (TMDs) for vibration suppression in both linear and nonlinear beam models, considering varying numbers of TMDs and the presence of internal damping. Particle Swarm Optimization (PSO) was consistently employed to ascertain the optimal positions, stiffnesses, and damping coefficients for all TMD configurations, with the objective of minimizing the area under the beam’s response curve.

Initially, a linear beam model without internal damping was analyzed. The optimization for a single TMD demonstrated a significant decrease in the beam’s vibration response. Expanding this to a two-TMD system also showed a significant attenuation compared to a single TMD. Further extending to a three-TMD system revealed an additional reduction in vibration when compared to the two-TMD setup. With internal damping introduced into the linear beam model, optimization of both single and two-TMD systems still led to effective vibration control. The three-TMD system provided an additional attenuation over the two-TMD setup in this internally damped environment. The investigation then progressed to the nonlinear beam model. For the nonlinear beam, a single optimized TMD again yielded a significant decrease in vibration. Introducing a two-TMD system for the nonlinear beam resulted in a significant attenuation compared to the single TMD configuration. Finally, the three-TMD system for the nonlinear beam showed a further attenuation when compared to the two-TMD setup. When internal damping was incorporated into the nonlinear beam model, the single and two-TMD configurations continued to provide effective vibration control. However, the addition of a third TMD yielded no further attenuation beyond that achieved with two absorbers, confirming that the optimal configuration for the this nonlinear damped system is the two-TMD setup.

A detailed analysis of the optimal TMD positions revealed that, regardless of linearity or internal damping, the optimizer consistently placed the absorbers near the antinode of the first mode, the most strongly excited mode under the impulsive load. When two TMDs were used, they either both remained near that antinode or one shifted slightly to also influence higher modes.

Overall, this research demonstrates the effectiveness of multi-TMD systems in attenuating beam vibrations across linear and nonlinear models, and with or without internal damping. While increasing the number of TMDs generally leads to further vibration reduction, the degree of attenuation decreases, particularly when moving from two to three TMDs and when internal damping is present, suggesting a trade-off between increased complexity and practical vibration suppression efficiency. The optimal number of TMDs, therefore, depends heavily on the specific beam characteristics, the presence of nonlinearities, and the desired level of attenuation. While this work establishes a strong foundation for controlling nonlinear vibrations with MTMDs under impulsive loads, future research will explore more complex loading conditions, such as moving loads and a wider range of excitation frequencies, to further generalize the findings.

## Data Availability

The datasets used and/or analysed during the current study are available from the corresponding author on reasonable request.
